# A Narrative Review of Alcohol Prevention Interventions Among Older Adults

**DOI:** 10.35946/arcr.v45.1.10

**Published:** 2025-09-30

**Authors:** Alexis Kuerbis, Silke Behrendt, Alex Elswick, Amy F. Kostelic, Simone Schultz

**Affiliations:** 1 Silberman School of Social Work, Hunter College, City University of New York, New York, New York; 2 Department of Psychology, University of Southern Denmark, Odense, Denmark; 3 Unit of Clinical Alcohol Research, Institute of Clinical Research, University of Southern Denmark, and Psychiatric Department, Region of Southern Denmark, Odense, Denmark; 4 Martin-Gatton College of Agriculture Food and Environment, School of Human Environmental Sciences, University of Kentucky, Lexington, Kentucky; 5 H.A.R.M.O.N.Y. Mental Health Counseling Services, Brooklyn, New York and Social Welfare, The Graduate Center, City University of New York, New York

**Keywords:** alcohol, prevention intervention, older adults, alcohol use disorder, primary prevention, secondary prevention, tertiary prevention, generation

## Abstract

**PURPOSE:**

Alcohol use and alcohol use disorder (AUD) among adults age 50 and older are an expanding public health challenge, requiring effective alcohol prevention interventions. Empirical literature on prevention interventions among older adults is limited by design issues, lack of publication, and misconceptions of aging. To enhance scientific rigor, prior reviews of prevention interventions among older adults excluded pre-to-posttest studies and studies with subgroups, such as veterans, racial minorities, and individuals who seek out digital interventions. The current narrative review aims to understand with whom prevention interventions for older adults are tested; describe barriers and facilitators of successful interventions; and include perspectives of both older adults and intervention providers. Unlike prior reviews, it includes a range of study designs, including digital interventions, and examines decade of age, periods in which studies took place, and generational factors associated with prevention intervention success.

**SEARCH METHODS:**

In December 2024, Boolean search terms, such as “alcohol*,” “older adults,” and “intervention,” were used across medical and social science databases, including PubMed, World of Science, PsycInfo, Social Sciences Citation Index, Cochrane Database, and other sources. The searches identified 983 articles published between 1999 and 2024, 582 of which were duplicates. Of the 401 abstracts reviewed, 231 did not mention older adults and/or alcohol. Thus, 170 full texts were reviewed. To be included, studies had to be peer-reviewed; have a mean participant age of 55 and older or a labeled subsample of individuals age 50 and older; focus on a nonpharmacological intervention; and reported alcohol or alcohol-related outcomes or older adult and/or provider perspectives of interventions. Studies set in a formal substance use treatment program were excluded. Overall, 84 records describing 51 interventions and 16 articles of consumer and provider perspectives were synthesized.

**SEARCH RESULTS:**

Studies were categorized into primary prevention, secondary prevention of AUD, and tertiary prevention of worsening AUD. Most interventions were delivered in person, in primary care, with individuals born from 1901 to 1923 (Greatest Generation) and 1924 to 1945 (The Silent Generation), and yielded significant reductions in alcohol use and related consequences. Only The Silent Generation consistently responded to interventions, demonstrating large effects. Additionally, two out of 18 randomized controlled trials found that individuals born from 1946 to 1964 (Baby Boomers) significantly responded to prevention interventions. Digital interventions were successful across generations.

**DISCUSSION AND CONCLUSIONS:**

Barriers to successful interventions occur at the organizational, provider, and older adult levels. Prevention intervention facilitators include drink tracking, agreement with another person, and aligning tone of the intervention to older adult perspectives of their drinking and perceived need to change. Adapting prevention interventions to older adults could include tailoring to an individual’s identity, culture, and meaning behind their drinking, which is often defined by generation, rather than only by age.

As the global population ages, alcohol use and alcohol use disorder (AUD) among older adults (defined here as adults age 50 and older) present a complex and growing public health challenge. Beginning at around age 50, adults experience physiological changes to the body and brain that affect their ability to metabolize alcohol, such as loss of lean body mass and an increasingly permeable blood brain barrier.^1^ These and other changes lead to greater intoxication and harm at lower levels of alcohol consumption, as well as to hazardous medication interactions and diminished ability to detect impairment. According to the Dietary Guidelines for Americans^2^ and the World Health Organization,^3^,^4^ low-risk drinking among older adults is defined as one standard drink (14 g ethanol) or less per day for women of any age and men age 65 and older, and as two standard drinks or less per day for men younger than age 65. In the United States, alcohol use among older adults has steadily increased over the past 20 years, with studies demonstrating an increase of up to 22% for binge drinking (five or more drinks for men, or four or more drinks for women, in a drinking episode^5^) and heavy drinking (drinking beyond low-risk guidelines^5^).^6^ Currently, 61.5% of U.S. older adults drink alcohol, 17% binge drink, 5% binge drink on five or more days in the past month, and 7% meet criteria for AUD.^5^ Strong associations between alcohol use in older adults and cancer, multimorbidity, hospitalizations, early nursing home entry, and mortality^7^ indicate that effective prevention interventions may be an important tool for addressing this public health problem.^6^

## Alcohol-Related Prevention Interventions Among Older Adults

Given the heightened vulnerability to alcohol among older adults, what should be the goal of prevention—abstinence (i.e., no alcohol consumption), moderate drinking (e.g., drinking at recommended low-risk levels), or harm reduction (e.g., any reduction in drinking to reduce risk of health or social consequences)?^7^ Existing prevention interventions are tailored to settings and individuals with goals ranging from abstinence to low(er) risk drinking. Policy initiatives and public health awareness campaigns to address other public health problems (e.g., anti-tobacco and opioid awareness campaigns) aim to reach the widest audience,^8^,^9^ yet most alcohol prevention interventions occur at the individual level, such as Screening, Brief Intervention, and Referral to Treatment (SBIRT) approaches.^10^ Designed for individuals whose alcohol consumption exceeds low-risk guidelines, including those with AUD, SBIRT is the predominant prevention intervention across the life span and set mainly in primary care.^11^ Its goal is to prevent and reduce harm across the drinking spectrum and to connect people who do not respond to the intervention to specialty treatment.^12^ SBIRT was disseminated in the United States via federal grants awarded to training programs, states, and academic hospitals to train providers to implement SBIRT.^13^ Early findings were promising,^11^,^12^,^14^ yet later meta-analyses found mixed results for SBIRT’s efficacy across settings, severity of alcohol use, and demographics.^15^,^16^ Additionally, there was a lack of data on SBIRT with older adults,^15^ and studies found an increasingly lower likelihood of alcohol screening as individuals aged.^17^–^20^ Subsequent grants were awarded to providers focusing on older adults,^10^ and SBIRT was adapted for older adults primarily by adding an age-specific health workbook^21^ for patients and providers to guide interventions. These initiatives have served thousands of older adults,^10^ yet their evaluation has been limited. Outcome data were not collected consistently; interventions were vaguely described; few studies used fidelity measures or reported intervention dosage; the effectiveness of individual components of the prevention interventions was rarely examined; and findings were often not peer-reviewed or published. When findings were reported, they often were limited to small subsamples and pre-to-posttest effects only, obfuscating older adult prevention intervention effectiveness.^22^,^23^

## Limitations of Prior Reviews of Prevention Interventions for Older Adults

Five systematic reviews^24^–^28^ have evaluated prevention interventions for alcohol use among older adults, and a sixth systematic review focused on digital alcohol interventions assessed age (i.e., age 55 and older) as a moderator.^29^ These reviews concluded that prevention interventions provide a potential for reduced harm among older adults; however, the investigators hesitated to draw definitive conclusions given unclear intervention descriptions,^24^,^26^ an absence of rigorous research design,^25^–^28^ and inconsistent outcomes^24^ within and across studies. To enhance methodological rigor, the reviews also excluded a substantial proportion of the empirical literature, such as pre-to-posttest designs and program evaluations, and key subgroups of older adults (i.e., veterans, inpatients, and racial/ethnic minorities),^28^ limiting generalizability. Other than the meta-analysis^29^ of digital interventions, which found that adults age 55 and older were 66% more likely to respond to online treatment compared to younger adults, no review included digital interventions.

In addition, none of the reviews above addressed potential generational effects on prevention interventions for older adults, despite the fact that generational cohort has often been cited as the primary cause of the dramatic increase in alcohol use among older adults described above.^6^,^30^ In 2025, adults ages 50 and older span multiple generations, The Silent Generation (TSG, which comprises individuals born from 1924 to 1945 who are now ages 80 to 101), Baby Boomers (BBs, born from 1946 to 1964, who are now ages 61 to 79), and Generation X (Gen X, born from 1965 to 1980, who are now ages 45 to 60). While alcohol remains the most used substance across older adult cohorts,^5^ each generation has experienced distinct historical and cultural contexts that have and continue to influence alcohol use after age 50. For example, average daily alcohol consumption decreased for the first part of the 20th century, with data showing that 50% of males from the Greatest Generation (GG, individuals born from 1901 to 1923) who drank reduced their consumption to moderate drinking (24 g of alcohol or less per day) starting around age 58, whereas in the following generation, TSG, this reduction occurred by age 46.^31^ Born during alcohol prohibition and temperance movements in the United States, TSG individuals eschewed heavy drinking and tended to view alcohol use as immoral.^32^,^33^ In contrast, alcohol use increased again among older adults as BBs began turning 50 in 1996.^34^,^35^ Characterized as a rebellious generation, BBs enjoyed greater access to and permissive attitudes toward drugs and alcohol with longer life expectancies, leading to at-risk drinking (i.e., drinking beyond low-risk guidelines) into their 60s and beyond.^7^

Age effects (i.e., where a particular age range or decade of life causes differential responses to interventions), period effects (i.e., when a particular era influences people across all age groups), and generation effects (i.e., a particular cohort of individuals born in a particular period) may all differentially influence preferences for and responses to prevention interventions. Generally, research to date mostly has assumed that aging/age, and its concomitant life events and physiological developments, is the most important factor in defining older adults as a group when tailoring interventions. Period effects likely also influence intervention preferences, such as the occurrence of a pandemic that necessitates remote health care as a new treatment and prevention approach; however, period effects can be difficult to parse out, especially in the absence of data across several decades. In contrast, generational effects are easier to identify and can address contextual factors of individuals born in a particular era. For example, Gen X, who began entering their 50s in 2015,^36^ is the first generation to grow up with computers and may be more receptive to digital prevention interventions compared to prior generations. Since generation is relatively unexplored as a factor in prevention interventions for older adults, an examination of if and/or how generation influences responses to alcohol prevention interventions is justified.

## The Current Study

This narrative review synthesizes literature on alcohol-related prevention interventions among older adults. It aims to understand how and with whom older adult prevention interventions have been tested, describe barriers and facilitators of successful prevention interventions, and collect older adult and provider perspectives to identify potential intervention adaptations in the future. It differs from previous reviews by: (1) including qualitative studies, pre-to-posttest designs, and quasi-comparison designs, in addition to randomized controlled trials (RCTs) to understand the state of prevention intervention research in older adults; (2) including digital interventions; and (3) examining studies in their historical context by investigating the generation(s) included and the years during which data were collected.

## Methods

**Figure 1:**
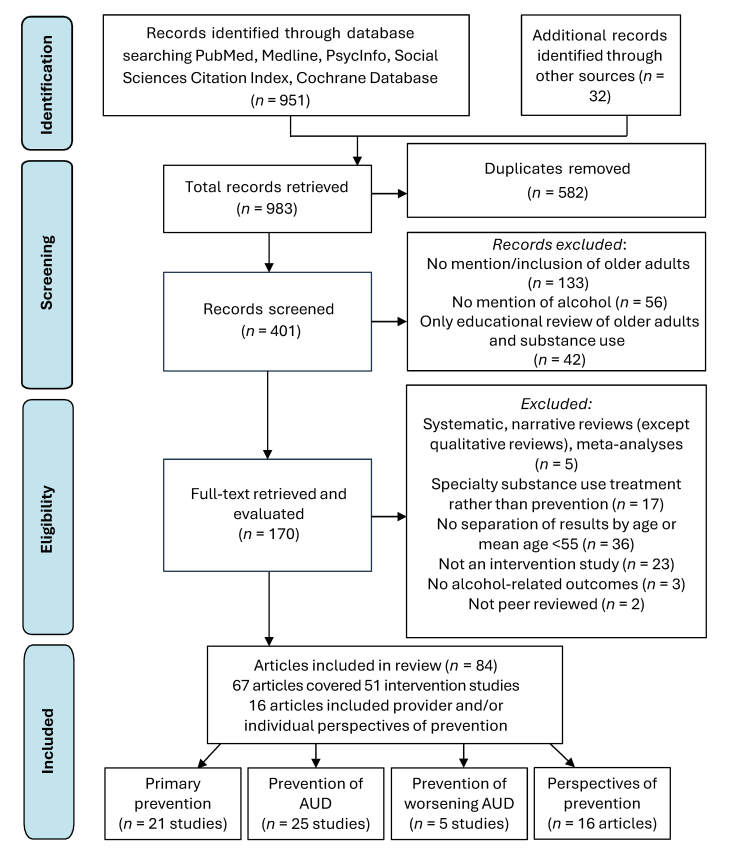
PRISMA diagram of review procedures. *Source:* Moher D, Liberati A, Tetzlaff J, et al. Preferred reporting items for systematic reviews and meta-analyses: The PRISMA statement. *PLOS Medicine* 2009;6(7):e1000097. doi:10.1371/journal.pmed.1000097

The literature search was conducted in December 2024 according to the Preferred Reporting Items for Systematic Reviews and Meta-Analyses (PRISMA) guidelines of systematic reviews (see Figure 1).^37^ Searches occurred across multiple databases, including PubMed, Google Scholar, World of Science, Cochrane Database, Social Sciences Citation Index, and PsycInfo. Boolean search terms—including “alcohol*,” “drinking,” “alcohol use,” or “alcohol outcomes”; “older adults,” “elderly,” or “seniors”; “intervention” or “brief intervention,” “prevention,” and “SBIRT”—were used in different combinations. Ancestry methods identified additional studies from study reference sections absent from the above-described searches. Studies were required to be peer-reviewed and published, although this requirement excluded major older adult prevention initiatives. A total of 983 records published from 1999 to 2024 were identified. After removing duplicates, 401 abstracts were screened for a focus on: (1) older adults or an adult population that typically includes substantial numbers of adults ages 50 and older (e.g., veterans); (2) alcohol use or a related outcome; and (3) a psychosocial intervention. Full texts were retrieved for 170 records.

Studies were included in the review if they: (1) had a mean age of 55 and older or a labeled subsample of individuals age 50 and older; (2) reported intervention outcomes or described the perspectives of older adults and/or providers on prevention interventions. Full texts were excluded if they: (1) were quantitative systematic reviews, meta-analyses, or reviews of reviews (qualitative systematic reviews were retained); (2) were not peer-reviewed; (3) covered pharmacological interventions; (4) described interventions set in a formal, specialty substance use treatment program; or (5) excluded alcohol outcomes. One study was excluded because its reported results conflicted with its conclusions.^38^ This resulted in 84 records (comprising 51 studies) on outcomes of alcohol prevention interventions and an additional 16 articles on participant and provider prevention intervention experiences.

Data extraction included sample description (size, mean/median age; race/ethnicity, and sex); years of data collection to identify generational cohorts; study design, setting, and intervention modality/approach; description of the intervention and its comparator; primary outcomes assessed; and findings. Outcomes of alcohol use (i.e., reported quantity and/or frequency of drinking) and alcohol-related targets (i.e., risk level, consequences of alcohol use, completed screenings, planning for change) were specific to each study. Intervention studies were sorted into three categories, relating to public health definitions of primary (i.e., disease prevention), secondary (i.e., early disease detection and intervention) and tertiary prevention (i.e., reduction in disease severity).^39^ This included primary prevention studies (*n* = 21), secondary prevention studies of AUD (*n* = 25), and tertiary prevention of worsening AUD severity (*n* = 5). Within each prevention category, results were organized by study design (i.e., pre-to-post, comparison designs [randomized or quasi-comparison], and RCTs) as a proxy for study bias and rigor. Prevention intervention barriers and facilitators were identified from all 84 articles and synthesized.

## Results

### Overall Study Characteristics

Table 1 summarizes characteristics of the 51 intervention studies.

These studies collected data from 1993 to 2021 and included participants from four generations: GG (included in 31% of studies), TSG (96% of studies), BB (57% of studies), and Gen X (14% of studies). None of the studies had a sample consisting predominantly (greater than 68%) or exclusively of GG participants. Among RCTs, TSG was the predominant generation in 75% of studies; BB participants were included with other generations in 25% of studies and were the predominant generation in 12% of studies. No study included a predominance of Gen X participants.

Most studies had samples with primarily White participants, with a third including more than 24% non-White participants. Almost half (47%) had samples with a majority of females, although males historically drink more and therefore would be expected to be more likely to participate. The most common intervention setting was primary care (45%), with a fifth delivered in a community agency and another fifth online. Prevention interventions were largely provided in person, some with a follow-up telephone or mail component. Some interventions were completely delivered remotely by mail, online, a smartphone app, or text messaging. Two-thirds of the interventions were adapted specifically for older adults. Interventions used mostly “motivational approaches” (77%), such as motivational interviewing,^40^ motivational enhancement therapy,^41^ SBIRT,^42^ and/or a feedback, responsibility, advice, menu of options, empathy, and self-efficacy (FRAMES) approach.^43^ Only three studies measured intervention fidelity.^44^–^46^ A quarter of the studies provided psychoeducation, and one-fifth used normative and/or personalized feedback. Among the 61% of studies that reported the number and duration of contacts with participants, about half occurred in one contact, ranging from 5 to 60 minutes, and half had multiple contacts (ranging from a total of 1 hour to 16 or more hours).

Focal outcomes were alcohol-related (e.g., completed screens; treatment engagement; reducing alcohol-related problems, harm, or risk level for consequences) and/or alcohol consumption (i.e., quantity, frequency of drinking, proportion of at-risk drinking). For alcohol-related outcomes, all prevention interventions successfully impacted their target outcome at intervention end. For alcohol consumption, the findings were mixed. Half of the interventions reduced the quantity and/or frequency of alcohol use (measured using the Alcohol Use Disorders Identification Test-Consumption [AUDIT-C]^47^ or Timeline Followback).^48^ Additionally, 79% of interventions reduced harm, such as alcohol-related consequences or behaviors as measured by the AUDIT,^49^ the Alcohol-Related Problems Survey (ARPS),^50^ its descendant the Comorbidity-Alcohol Risk Evaluation Tool (CARET),^51^ and the Alcohol, Smoking, Substance Involvement Test (ASSIST).^52^ Notably, many RCTs reported significantly reduced alcohol use across conditions, including control conditions or active comparators, potentially indicating that assessment itself had a therapeutic effect among older adults.

### Primary Prevention Interventions

#### Pre-to-posttest designs

Of the 12 primary prevention interventions evaluated using pre-to-posttest designs (see Appendix 1), 11 resulted in significant increases in knowledge, completed screening, intention to change or talk to a professional, and decreases in alcohol use and risk level, where measured. Studies were mostly set in primary care offices and senior centers, with interventions ranging from simple brochures to class-like formats; sample sizes ranged from 26 to just under 18,000 participants. Studies are organized by their target outcome below.

##### Increased knowledge as outcome

All five studies aimed at increasing knowledge of alcohol-related risks reported significant increases in knowledge immediately after the intervention.^53^,^54^,^56^,^57^,^59^ Three of the studies^53^,^56^,^57^ provided in-person group education in a senior center, with two^53^,^57^ (one with GG/TSG participants, one with TSG/BB participants) utilizing a class-like format. Only one of these two studies demonstrated sustained knowledge at 6 months, with slippage in some areas.^57^ A third study implemented an intervention called *Prevention BINGO* with 348 TSG/BB participants.^56^ Gameboard squares were facts about alcohol use and relevant risks, and participants earned prizes. Participants’ increased knowledge was sustained 1 month later. Two other studies^54^,^59^ offered effective one-on-one education—one delivered it via a 36-page booklet or pamphlet to GG/TSG participants in a primary care waiting room,^54^ the other via a short video public service announcement, a poster, and a brochure to TSG/BB participants at a pharmacy.^59^,^62^ In the primary care setting, participants preferred the booklet over the pamphlet;^54^ in the pharmacy setting, participants preferred all three mediums together.^59^

##### Intention to talk with a professional about drinking as outcome

Two studies tested participants’ immediate intention to discuss alcohol use with a professional after reviewing educational materials.^55^,^59^ Among GG/TSG older adults who completed the ARPS^78^ and received older adult-specific feedback in a primary care waiting room, 31% intended to talk to their primary care physician about their alcohol use.^55^ A second study in a pharmacy with TSG/BB older adults increased knowledge, but participants reported no intention of discussing drinking with a pharmacist.^59^

##### Completed screening as outcome

One study demonstrated a threefold increase in alcohol screening among TSG/BB older adults when screening was included in an online Medicare annual wellness visit.^67^

##### Plan for change as outcome

Four studies^20^,^57^,^58^,^62^,^63^ used plan for change post-intervention as a proxy outcome. The online service Alcoholscreening.org assesses visitors’ alcohol consumption through a brief assessment and provides both normative feedback (comparison to peers’ drinking) and personalized feedback (risk-related feedback).^58^ In 2013, TSG/BB visitors (*n* = ~17,600) to the site had significantly (~50%) higher odds of initiating a plan for change compared to those under age 50. In another study comparing types of online-delivered older adult-specific feedback (normative vs. personalized feedback) with BB/Gen X older adults, 44% of participants across conditions planned to reduce drinking.^63^ A subsequent analysis showed that older men receiving normative feedback were more likely to plan for change.^79^ Another study implemented a 5-minute, in-person brief intervention in atypical settings (e.g., grocery stores) with TSG/BB/Gen X older adults; 40% of older adults drinking beyond low-risk guidelines intended to change.^20^ Finally, the previously mentioned pharmacy-based study with TSG/BB older adults that implemented multipronged psychoeducation did not increase intention to change drinking.^59^

##### Reduced at-risk drinking as outcome

Two studies^65^,^66^ tested SBIRT delivered by nonphysicians with TSG/BB older adults, both of which included a relatively high proportion of non-White participants. One study sample had 12% participants age 65 and older and 37% non-White participants.^66^ At 6 months, those age 65 and older had the lowest intervention response of any age group, showing an 11% reduction in at-risk drinking compared to 20% among younger adults. The other study sample included only adults age 60 and older, of whom 95% were African American.^65^ The intervention was tailored to older adults, and findings demonstrated a 65% reduction in at-risk drinking at 6 months.^65^ The study’s intentional use of participants’ long-established relationships with health care providers may be a reason for its success. A third study—the pharmacy-based psychoeducation with TSG/BB described above that did not increase intention to change—found that individuals in the sample who consumed alcohol beyond low-risk levels cut their drinking from 15 drinks per week to seven drinks per week 3 months later.^62^

#### Randomized comparison study

One study compared the effectiveness of two online interventions using increases in completed screening and access to further resources as outcomes.^68^ To increase online screening engagement, visitors to the drinkaware.co.uk website randomly received health- or appearance-based alcohol banner messages to determine which led to greater AUDIT-C^47^ screening completion. Banner messages appeared at the top of a computer or mobile phone screen, displaying either a message about how drinking affects one’s health or how drinking affects one’s appearance, such as premature aging.^68^ Among adults ages 45 to 64 (mostly BBs ages 50 to 68 and Gen X ages 45 to 49), both health- and appearance-based messages led to screening and accessing resources; however, for adults age 65 and older (mostly TSG), appearance-based messages led to significantly more completed screens.

#### Randomized controlled trials

##### Alcohol use and at-risk drinking as outcomes

Among seven RCTs testing primary prevention interventions for alcohol use that reported drinking outcomes,^70^–^77^ only one demonstrated a significant effect compared to usual care.^70^ Fink et al.^70^ implemented a primary care prevention intervention with GG/TSG older adults. After completing the older adult-specific ARPS,^80^ participants were randomized to one of three arms: (1) usual care; (2) a feedback and education report given to the participant; or (3) a report given to both the participant and the primary care physician (combined report). At 12 months, older adults in the patient-only report group were 59% more likely to drink at low-risk levels according to the ARPS compared with those who received usual care. With the combined report, participants were 23% more likely to drink at low-risk levels compared with those receiving usual care. However, it was only the combined report that demonstrated a significant, though small, reduction in standard drinks (just over one standard drink) at follow-up compared to usual care—the only significant finding in terms of alcohol consumption. In contrast, the same investigators later tested the effectiveness of an educational website to teach older adults to balance risks and benefits of alcohol use among TSG/BB older adults outside primary care compared to no intervention;^76^ both groups reduced drinking by about two drinks per week.

##### Treatment engagement as outcome

The Healthy Profiles Study^69^ compared a brief alcohol intervention booklet to a general health advice booklet provided within primary care among TSG male veterans. Alcohol use outcomes could not be ascertained, but the alcohol booklet increased participants’ use of outpatient specialty care compared to the general health booklet at 9 months. No other differences were reported.

##### Healthy lifestyles as outcome

Five RCTs of interventions that aimed to improve healthy lifestyles among GG/TSG older adults or recruited TSG/BB older adults coping with health problems or events (e.g., hospitalization, emergency department visit, bone density scan, stroke patients) found that the interventions were not effective in significantly reducing alcohol use.^71^–^73^,^75^,^77^ Intervention modalities varied across studies (four were delivered in person, three involved printed materials, one was delivered online). Two RCTs addressing overall lifestyle among GG/TSG older adults^71^,^72^ intervened with the participants or their primary care physician separately. Neither approach reduced alcohol use at the 1-year follow-up.

#### Summary of findings from primary prevention studies

Existing primary prevention interventions among older adults increased knowledge about alcohol use and its potential harms in later life in pre-to-posttests,^53^,^54^,^56^,^57^,^59^,^62^ increased both initiation and completion of screens for alcohol use,^67^,^68^ prompted intention to talk to a physician^68^ (but not a pharmacist^59^) about drinking, and prompted planning or intention to change.^20^,^58^,^63^ Within-group studies of TSG/BB older adults revealed that SBIRT delivered by a nonphysician yielded a lower response among those age 65 and older compared to younger adults; however, a tailored, older adult-specific SBIRT intervention in primary care settings and senior centers significantly reduced alcohol use across time, with AUDIT-C measured risk identified in fewer and fewer cases across time.^65^ Only 1 out of 7 primary prevention RCTs demonstrated significant decreases in at-risk drinking compared to the control condition,^70^ an effect that was strongest when only participants and not their physicians received the report on the alcohol assessment. This RCT took place from 2000 to 2003among GG/TSG older adults. Since then, no other RCT has demonstrated this same effect for a primary prevention intervention. RCTs that found no significant effects were focused on overall lifestyles, multiple target behaviors, or implemented among individuals experiencing severe health issues. Participants in acute care may have been too ill or compromised to respond to the interventions,^75^,^77^ and passive feedback^73^ may have been ignored altogether. A few studies focused on engaging older adults in screening or education, which led to innovations such as gamification^56^ and alternative messaging.^68^

### Secondary Prevention Interventions for AUD and Alcohol-Related Problems

Of 25 secondary prevention interventions for AUD assessed (see Appendix 2), four were assessed using pre-to-posttest designs,^22^,^23^,^81^–^83^ one with a randomized comparison trial,^84^ and 20 with RCTs.^44^,^85^–^91^,^93^–^97^,^99^,^100^,^102^–^113^ Studies focused on mostly male GG/TSG older adults, implementing interventions using multiple, longer contacts, and 62% were older adult-specific. A majority were set in primary care (56%), followed by online (20%).

#### Pre-to-posttest designs

All four pre-to-posttest studies^22^,^23^,^81^–^83^ reported significant reductions in alcohol use after the intervention among older adults with at-risk drinking (see Appendix 2). Three interventions were delivered in person;^22^,^23^,^81^,^82^ the fourth was online.^83^ U.S.-based study samples had about 20% non-White participants. Studies are summarized in chronological order below.

The Florida Brief Intervention and Treatment for Elders (BRITE) Project^22^,^23^ was implemented in two phases—a pilot phase and a larger SBIRT initiative—implemented from 2004 to 2011. Across both phases, 88,498 GG/TSG/BB older adults were screened in community settings and participants’ homes by health educators, with 11,663 individuals screening positive for substance misuse. Screening assessed alcohol use (pilot: AUDIT^49^ and Short Michigan Alcoholism Screening Test-Geriatric Version [SMAST-G];^114^ larger study; ASSIST^52^), depression (Patient Health Questionnaire-2^115^,^116^ and Geriatric Depression Scale, Short Form^117^), and/or anxiety (pilot: taking anti-anxiety medication^23^). Participants screening positive for alcohol misuse were offered SBIRT, with the option of up to five 1-hour sessions in Phase 1 or up to 16 or more sessions in Phase 2, including motivational interviewing and, if needed, older adult-specific cognitive behavioral therapy modules.^10^ Health educators determined the number of sessions needed for an individual, with most ending without implementing cognitive behavioral therapy.^10^ In Phase 1, only 19% of the 169 GG/TSG participants with at-risk drinking at baseline remained positive for at-risk drinking 30 days post-discharge.^23^ In Phase 2, TSG/BB participants with 6-month data reported a reduction in any alcohol use (45% of participants), having five or more drinks in one sitting (23% of participants), and drinking to intoxication (10% of participants).^22^ Results should be interpreted cautiously given that out of the 6,600 participants who received services only 171 completed follow-up.

The Older Wiser Lifestyles intervention^81^,^82^ adapted the ARPS for Australian TSG older adults age 60 and older who drank alcohol in the past month. Those who screened positive for at-risk drinking were invited to a primary care clinic for an in-person, stepped-care intervention. Older adults with low-risk drinking were provided minimal education. Older adults with at-risk drinking were assessed for readiness to change, and brief interventions were tailored accordingly. Those in the precontemplation stage received a 1-hour session of psychoeducation; those in the contemplation stage received up to 5 hours of motivational interviewing and cognitive behavioral therapy. Over half (54%) of those at-risk drinking received just one session, with participants reporting it was sufficient.^81^,^82^ AUDIT-C scores were significantly reduced at the 6-month follow-up, yet 36% of participants continued at-risk drinking.

Lastly, a smartphone app adapted a cognitive approach bias modification intervention for BB/Gen X Australians.^83^ Older adults who drank beyond low-risk guidelines trained on the app twice per week for 4 weeks using their personal smartphone. At-risk drinking decreased for both for single sittings (down 16%) and total weekly consumption (down 22%) after the intervention, with a mean reduction of consumption of 5.4 drinks per week.

#### Randomized comparison study

Sedotto et al.^84^ randomly assigned adults to one of two digital, non–age-specific interventions: Step Away, an eight-module, smartphone app-based intervention and a Facebook chatbot (see Appendix 2). The app required participant initiation, whereas the chatbot was less formal and initiated daily check-ins. Significant alcohol use reduction occurred across conditions and age groups; overall, there was a reduction in consumption of about 1 drink per drinking day and a 28% increase in days abstinent. However, BB/Gen X older adults engaged twice as often as younger adults across the interventions to achieve this reduction. Older adults may need greater digital intervention exposure for equivalent therapeutic effect.

#### Secondary prevention interventions tested with RCTs

RCTs addressed at-risk drinking among only older adults (*n* = 18) or compared them to younger adults (*n* = 2) (see Appendix 2). Fifteen interventions were older-adult specific.^85^,^86^,^88^–^91^,^93^–^97^,^99^,^100^,^102^,^104^–^110^,^113^ Fourteen studies were conducted in primary care;^85^–^88^,^90^,^91^,^93^–^97^,^99^,^100^,^102^,^104^–^107^,^109^,^110^,^113^ additional settings included community agencies, inpatient and outpatient hospital units, and online. Sixteen RCTs were at least partly in-person;^44^,^81^,^82^,^87^,^89^,^93^–^97^,^99^–^101^,^104^–^107^,^109^,^110^,^112^ five had follow-up telephone calls.^44^,^89^,^96^,^97^,^99^–^101^ Primary outcomes were alcohol use, proportion of people with at-risk drinking, treatment engagement, and health care utilization. Six RCTs reported significant effects in favor of prevention intervention compared to controls.^85^,^88^,^96^,^99^,^108^,^111^ Seven studies had mixed findings, with significant results in favor of the prevention intervention only for a sample subgroup.^90^,^91^,^93^–^95^,^103^,^110^,^118^ Eight RCTs found no effect across conditions.^44^,^87^,^89^,^91^,^104^,^106^,^107^,^109^,^112^

##### RCTs with significant positive effects

Project Guiding Older Adult Lifestyles^85^ tested a primary care-based alcohol prevention intervention tailored to GG/TSG compared to usual care. The intervention consisted of two 10- to 15-minute counseling sessions (1 month apart) with a primary care physician, during which participants received brief advice and psychoeducation on drinking risks, reviewed an information booklet, and contracted for change. Weekly alcohol use declined by 36% and binge drinking by 47% in the intervention group compared to control at 12 months,^85^ and this decline was sustained 24 months later.^86^ Generalizability of findings was limited because only 35% of participants were female and other demographics were not reported.

Another study randomized mostly male, non-White TSG veterans with depression and/or at-risk drinking to usual care or telephone disease management plus usual care.^88^ Telephone disease management included six calls with a behavioral health specialist nurse, during which participants completed an older adult-specific workbook to reduce drinking, for a total of 4.5 hours over 24 weeks. Follow-up occurred at 4 months, before intervention end. Telephone disease management plus usual care was superior to usual care alone in treatment engagement (43.8% vs. 20%, respectively), reduction in drinks per week (9 units vs. 2 units, respectively), and reduction in binge episodes over a 3-month period (26 episodes vs. 1 episode, respectively).^88^

The Healthy Living as You Age study^96^,^97^ focused on TSG/BB older adults with at-risk drinking; the sample included 22% Hispanic or non-White individuals, and 29% were female. All participants were screened using the CARET,^51^ an ARPS descendent, and then randomized to receive a general health booklet or a prevention intervention. The intervention consisted of a daily diary of alcohol use before a primary care visit, primary care brief advice using an older adult-specific booklet, and three sessions of motivational interviewing with a health educator, totaling 80 minutes. At-risk drinking was significantly reduced at 3 months in the intervention group compared to controls.^96^,^97^ Although a reduction was sustained at 12 months, only past-week drinking remained significantly different from control.^96^ Older adults who received all three health educator calls had more than five times greater odds of not exhibiting at-risk drinking compared with those who received no calls at 3 months but not 12 months.^97^

Project Share^99^,^100^,^102^ randomized TSG older adults to usual care or an intervention that included mailed psychoeducation booklets/tip sheets, a daily diary of drinking before a primary care visit, a discussion with a primary care physician, and three health educator calls, totaling 27 minutes. Primary care physicians were asked to discuss alcohol use at each visit for a year. If a physician discussion occurred, odds of at-risk drinking at 12 months decreased by 39%, and if both a daily diary and agreement to reduce drinking were completed, odds decreased by 55%.^100^

Two prevention interventions, one mail-based^108^ and the other text messaging-based,^111^ demonstrated significant effects among older adults. TSG/BB older adults from a primary care network identified as exhibiting at-risk drinking were randomized to receive either mailed brief personalized feedback (based on the CARET) with booklets on alcohol and aging^119^ or assessment only.^108^ At 3 months, the intervention group significantly reduced at-risk drinking by 22%, alcohol medication co-use by 15%, and symptoms of medical or psychiatric conditions by 20% compared to the control group. Using secondary analysis, another study examined pilot RCT data comparing 12 weeks of non-age-specific daily text messaging to assessment only to reduce at-risk drinking.^111^,^120^ BB adults age 50 and older were compared to younger adults. Text messaging reduced drinking by about 5 units per week and 1 unit per day across age groups by 3 months compared to assessment only. Younger adults had slightly larger effects than older adults. Older adults had a stronger response to gain-framed messages (e.g., messages about the benefits of reducing alcohol use), whereas younger adults responded more to loss-framed messages (e.g., messages about the negative consequences of drinking).

##### RCTs with mixed findings on alcohol use

Five studies^90^,^91^,^93^–^95^,^118^ were part of the Primary Care Research in Substance Abuse and Mental Health for the Elderly (PRISM-E) project. PRISM-E compared “integrated care,” defined as combined mental health and substance use treatment in primary care, to “enhanced referral to specialty care” on engagement with specialty treatment and alcohol use. Ten sites across the United States enrolled more than 2,000 TSG older adults,^90^ with 26% female and 48% non-White participants. Each site followed set guidelines for the conditions yet tailored protocol details to fit their site’s needs.^118^ In integrated care, all sites provided a brief intervention with an age-specific workbook. Some sites offered additional sessions after the brief intervention or comprehensive geriatric care of differing intensity and professional make-up.^95^ Treatment engagement was higher with integrated care (71% of participants) compared with enhanced treatment referral (49%); however, given intervention variability, determining the components responsible for this difference is complex.^90^ An analysis of three sites found no significant condition differences for alcohol use at 6 months; however, the authors noted that although 43% of participants in the integrated care group received at least one session of the brief intervention, only 9% received all three sessions offered at this particular site.^91^ A single site (*n* = 34), which emphasized harm reduction via three sessions of motivational interviewing within integrated care, reported that those assigned to integrated care were significantly more likely to access specialty care and reduce drinking compared to enhanced referral.^94^ Another analysis of 12-month data from male veterans with at-risk drinking within the PRISM-E project found reduced binge drinking.^93^ One site treating veterans implemented integrated care with an interdisciplinary team of geriatric specialists; this approach demonstrated a 75% reduction in the likelihood of at-risk drinking compared to enhanced referral.^95^ Overall, setting attributes, drinking severity, and intervention exposure contributed to distinct outcomes across sites. Thus, sites with more completed sessions and/or services^91^,^93^,^94^ within integrated care appeared to be associated with increased treatment engagement and reduced at-risk drinking compared to enhanced referral; notably, enhanced referral also performed well at these sites.

Some studies noted differences in response to the interventions by sex. A Danish RCT tested an online intervention among 1,380 TSG/BB older adults.^103^ Participants were randomized to one of three arms: personalized and normative feedback, brief advice, or a control. All groups reduced drinking by about 6 units per week at 12 months; however, males were found to respond significantly more to personalized and normative feedback than to the other two conditions.

A Spanish RCT evaluated in-person brief interventions in health centers and homes at four time points over 12 months compared to brief advice given at the first visit.^110^ Significant intervention effects were found only among women, who were four times as likely to have reduced drinking compared to controls.

##### RCTs with null findings

Five studies in primary care settings found no effects of the interventions.^44^,^87^,^104^,^106^,^109^ Three of the studies^44^,^104^,^109^ reported methodological issues, namely poor compliance, poor competence in intervention delivery, or low exposure to the intervention; however, two methodologically strong studies^87^,^106^ also found no condition differences. Cucciare et al.^106^ randomized mostly male TSG/BB veterans who screened positive for at-risk drinking to either web-based normative feedback (with brief advice) or usual care. At 12 months, both groups reduced heavy drinking days by 50%, reduced consumption by one drink per day, and had three fewer drinking days per month. Gordon et al.^87^ compared the efficacy of motivational enhancement treatment, brief advice, or usual care among adults drinking beyond low-risk guidelines. All groups, regardless of condition or age, significantly reduced drinking at 12 months, yet GG/TSG older adults age 65 and older still drank beyond low-risk guidelines.

Outside of primary care, two studies implemented prevention interventions with injured TSG/BB/Gen X older adults^112^ or ill GG/TSG patients^89^ in a hospital setting compared to usual care. Both studies had null findings, similar to primary prevention studies that targeted ill or injured older adults.^89^,^112^

#### Summary of findings from secondary prevention studies

Pre-to-posttests of secondary prevention interventions^22^,^23^,^81^–^83^ included multiple contacts with participating older adults and demonstrated significant reductions in at-risk drinking, averaging a reduction in alcohol use by about half across studies. Secondary prevention interventions tested with RCTs that demonstrated a significant therapeutic effect compared to the control or comparison group shared important features. All six of these studies^85^,^88^,^96^,^99^,^108^,^111^ focused on one outcome—reduced at-risk drinking—and all were focused on TSG individuals, with one study including GG older adults.^85^ Five of the six studies^85^,^88^,^96^,^99^,^111^ had interventions with clearly defined multiple points of contact, and four had a behavioral nurse specialist or physician involved.^85^,^88^,^96^,^99^ Three digital interventions,^84^,^111^ focused on TSG/BB and BB/Gen X individuals, significantly reduced drinking among younger and older adults. Older adults engaged more with interactive digital interventions^84^ and responded differentially to distinct text messaging types compared to younger adults.^111^ All three digital interventions had multiple points of contact, and no professional was involved. Secondary prevention interventions that yielded null findings were largely methodologically flawed,^44^,^104^,^109^ appeared to have limited treatment exposure, 91 or had only a single point of intervention contact.^87^

### Tertiary Prevention of Worsening AUD Outside of Formal Treatment

Five studies involved individuals with AUD outside specialty treatment. Two were quasi-comparison studies,^46^,^121^,^122^ two were randomized comparison trials,^123^–^125^ and one was an RCT^45^ (see Appendix 3). All tertiary prevention interventions were delivered in person, with mostly TSG/BB individuals, and had significant therapeutic effects. Two interventions were specifically designed for older adults.^121^,^123^,^126^ Four used motivational interviewing and/or cognitive behavioral therapy.^45^,^46^,^123^–^125^

#### Quasi-comparison studies

Both tertiary prevention interventions that were tested using comparison with no treatment without randomization reduced alcohol use.^46^,^121^ A single-system analysis examined 38 GG/TSG/BB individuals (age 54 and older) from three RCTs testing non-age-specific brief treatments for AUD.^46^ Older adults in active treatments reduced drinking twice as often as those receiving no treatment by the end of the treatment period. A second study aimed to increase discharges to home among older adults with substance use histories in a skilled nursing facility. The facility offered an onsite recovery program,^121^,^126^ including general/specialty counseling, family therapy, and 12-step meetings, with supportive home visits after discharge. Odds of discharge to home of individuals participating in the recovery program were three times those of individuals who did not participate in the program.

#### Randomized comparison and controlled trials

Three studies each aimed to test the impact of two active treatments.^45^,^123^–^125^ A Swedish study randomized TSG/BB/Gen X older adults to two active brief treatments pathways—the “15-method” or specialty alcohol treatment. Both conditions provided brief advice with a physician and pharmacological and/or psychosocial treatment for AUD.^124^,^125^ Neither treatment was specific to older adults, and the “15-method” was briefer than specialty care. At 12 months, alcohol use was reduced across groups, with no moderating effect of age, and participants preferred specialty care.^124^,^125^ Another study randomized TSG older adults either to a community-based/in-home prevention intervention providing geriatric care management, or to direct referral to enhance specialty treatment completion.^123^ The geriatric care management included motivational interviewing and multiple points of contact. Compared to direct referral to specialty care, the intervention group had approximately 30% higher rates of inpatient and outpatient treatment completion. Finally, data from two RCTs were combined to explore the effectiveness of four sessions of motivational interviewing versus four sessions of nondirective listening for 8 weeks across age groups.^45^ Older adults age 51 and older responded best to the high-quality motivational interviewing; however, this approach reduced drinking only 8% more than did nondirective listening by week eight.

#### Summary of findings from tertiary prevention studies

Tertiary prevention studies that offered more points of contact and services appear to be the most preferred, significantly reduced alcohol use, or increased treatment completion. When compared to no treatment,^46^,^121^,^122^ active interventions that included pharmacology and counseling demonstrated greater drink reduction. When active interventions were compared to one another,^45^,^127^ drinking outcomes were equivalent or demonstrated minimal difference.

### Intersection of Age, Period, and Generation

Table 2 lists the interventions that demonstrated at least preliminary effectiveness compared to another existing treatment or to control in the context of an RCT, organized by decade of age of participants, years of data collection, and the predominant generation included in the sample. Of 14 RCTs with positive findings (see Table 2),^45^,^69^,^70^,^85^,^88^,^90^,^93^–^96^,^99^,^108^,^111^,^123^ across decades of age, only three^45^,^108^,^111^ collected data before 2008. Those three completed data collection in or before 2016.

Among older adults ages 50 to 60, five RCTs^45^,^69^,^88^,^108^,^111^ with favorable results for the intervention were implemented with TSG or BB individuals. Two studies^108^,^111^ outside of primary care found preliminary intervention effectiveness among BBs ages 50 to 60 for secondary prevention only. In contrast, nine RCTs^44^,^76^,^77^,^103^,^104^,^106^,^107^,^109^,^112^,^124^,^125^ had null or equivalent findings among predominantly BBs ages 50 to 60.

Among older adults ages 61 to 70, 12 RCTs^45^,^69^,^85^,^88^,^90^,^93^–^96^,^99^,^108^,^111^,^123^ noted significant differences, including 10 with TSG individuals^69^,^85^,^88^,^90^,^93^–^96^,^99^,^123^ and two with TSG/BB individuals.^45^,^108^ Two primary prevention interventions^85^,^88^ and seven secondary prevention interventions^69^,^90^,^93^–^96^,^99^ with TSG older adults were set in or connected to medical settings and contained multiple points of contact. Two tertiary prevention studies with participants in this age group found a significant effect.^45^,^123^ One offered multiple services in the context of geriatric case management with TSG individuals,^123^ and the other found a small effect for motivational interviewing among BB individuals over nondirective listening.^45^ In contrast, six studies^75^,^87^,^89^,^91^,^104^,^110^ of predominantly TSG individuals and two with a combination of TSG/BB individuals^73^,^109^ in this age group found no significant effects, thought to be due to methodological issues and/or minimal intervention. One methodological exception among the null studies was a U.K. study set in primary care that provided stepped care for alcohol use compared to 5 minutes of brief advice with a nurse for TSG/BB individuals.^104^ Six studies with predominantly BB individuals found no significant effects.^44^,^77^,^103^,^106^,^107^,^112^,^124^,^125^

Among older adults ages 71 to 80, two primary prevention interventions took place in primary care, one with GG^85^ and one with GG/TSG individuals.^70^ Six secondary prevention interventions,^90^,^93^–^96^,^99^ four of which were part of PRISM-E,^90^,^93^–^95^ and one tertiary prevention intervention served TSG individuals.^123^ In contrast, seven studies^71^,^72^,^75^,^87^,^89^,^91^,^110^ with TSG individuals with null findings included ill or injured participants, minimal interventions, and/or had multiple target behaviors. No study included BB participants in their 70s.

### Barriers and Facilitators of Successful Prevention Interventions

The studies reviewed here identified numerous barriers to successful older adult prevention interventions (see the box “Potential Barriers to Successful Prevention Interventions Among Older Adults”). Organizational level barriers included limited resources, payment structures that discourage SBIRT, and lack of leadership commitment to prevention intervention sustainment beyond study end.^60^,^65^,^66^,^128^,^129^ In acute health care settings, prevention interventions failed to impact alcohol use because other health concerns may be more pressing.^75^,^77^ Further, interventions that target too many behaviors at once,^67^,^71^,^72^ are too passive (e.g., a letter),^73^ or have unverified or low exposure to intervention dose,^26^,^91^,^97^,^104^,^128^ were ineffective in producing alcohol-related behavior change. Qualitative studies found that providers who did not believe older adults drink alcohol and/or could change, only looked for AUD symptoms, or feared ruining rapport with older adults by asking about alcohol use, were reluctant or refused to implement prevention interventions.^60^,^130^–^132^ Providers also reported low motivation to screen due to limited time and little training to respond to positive screens.^60^,^129^–^132^ From their perspective, older adults reported feeling that providers did not want to hear about or treat drinking,^128^,^129^ that the topic was too personal to broach,^17^,^128^ or that the provider was too young to understand.^20^

When older adults perceived interventions to be irrelevant to their situation or misaligned with their preferences or circumstances, they were reluctant to or incapable of changing alcohol use before resolving their stress and mental health problems.^17^,^63^,^133^–^135^

**Table:** 

**Potential Barriers to Successful Prevention Interventions Among Older Adults**
***Organizational Factors***
No third-party reimbursement for intervention^60^,^66^
Cost and availability of staff to implement^65^,^128^
Cost and availability of additional help^65^,^128^
Low readiness to include regular screening, brief intervention, and referral to treatment (SBIRT) or other screening^65^
Low commitment to implement SBIRT or other screening^65^
High staff turnover^65^
Lack of resources (e.g., space, administrative support)^65^
Contractual agreements that inherently discourage assessment^129^
No on-site champions for screening and interventions, leadership^65^
***Intervention Factors***
Set in acute care settings where other health problems may be priority^75^,^77^
Too general or too many target behaviors^67^,^71^,^72^
Too passive (e.g., a letter with results for seemingly unrelated health problem)^73^
Unverified or low exposure to intervention^26^,^73^,^88^,^91^,^92^,^97^,^104^,^125^
Definition of standard drink without visual or experiential learning that does not hold^20^,^128^
***Provider Factors***
Perceptions of Older Adults
Older adults don’t drink^130^
Alcohol is permanently entrenched in life, no possibility of change^130^
No insight among older adults, in denial, health illiteracy^60^,^130^
Older adults are fragile, fear of being insensitive or ruining rapport^60^,^130^,^131^
Right to self determination^130^
Alcohol Only Needs to Be Focus of Concern When
Risk of harm in home environment (e.g., fire)^130^,^131^
There are alcohol use disorder symptoms present^130^,^131^
Low Motivation to Screen
Need to prioritize other chronic conditions^130^,^131^
Inadequate training, support and/or place to refer older adults to for specialized treatment^60^,^129^,^130^
Too busy, too little time, not interested^129^,^132^
Low job satisfaction to screen or treat^129^
Can’t directly observe improved quality of life of the older adults^130^
***Older Adult Factors***
Experience in Health Settings in General
Perceive primary care provider as not wanting to treat drinking; drinking is not legitimate issue to bring up^128^,^129^
Discomfort, embarrassment, feels too personal to discuss with primary care provider^17^,^128^
Provider is too young, won’t understand^20^
Self-Perception, Preferences, and Experience Misaligned with Intervention
Considers themselves as drinking responsibly, no need to change, regardless of risk information^133^,^134^
Consider themselves wiser due to age, can handle drinking^134^
Alcohol-related health salience (no apparent consequences, no change)^17^,^134^,^135^
Doesn’t consider information relevant, even if it includes their age group^60^,^128^,^133^,^136^
Believability of the education/feedback (doubt its veracity, not convinced)^63^,^128^,^129^,^133^,^136^
Feel pushed, preached to^128^
Does not want to be abstinent, prefers moderation^17^
Physical/mental health problems are more urgent, wants to treat those first^133^

Conversely, numerous attributes can enhance the success of prevention interventions (see the box “Facilitators to Successful Prevention Interventions Among Older Adults”). Primary prevention interventions implemented where older adults are waiting and otherwise unoccupied (e.g., waiting room, pharmacy, hospital atrium) provided opportunities to engage older adults and were acceptable across generation and sex.^20^,^54^,^59^–^61^,^70^ Flexibility of place, timing, and frequency of contact,^20^ such as including grocery stores, in-home visits, or other places older adults routinely visit (and outside an acute care setting), can better capture older adults’ attention and provide access to brief interventions for older adults. Playful activities, such as gamification of prevention interventions,^56^ may also engage older adults more effectively by broadening education and screening, normalizing alcohol conversations, and reducing stigma.^127^ Steps taken to increase trust in information provided to older adults, such as using established and trustworthy sources that also illustrated the relevance of information to their level of risk and perceived concern, appeared to better engage older adults in evaluating their drinking and planning for change.^63^,^79^,^128^,^129^,^133^,^136^,^137^ In primary care settings, prevention intervention components effective across generations included onsite behavioral health services, 90 drink tracking,^45^,^96^,^97^,^99^,^100^,^102^ agreements with another person to reduce drinking,^96^,^97^,^99^,^100^,^102^ normative and/or personalized feedback,^63^ full dosage of intervention,^97^ and follow-up visits or calls with providers or peers over months for further support and/or accountability. Physician involvement^69^,^96^,^98^–^100^,^102^,^125^,^138^ appeared crucial for older generations (GG/TSG), whereas provider profession appeared less pivotal for younger cohorts. Studies in which both physician and patient were provided with feedback were the most effective among GG/TSG older adults. Whether professionals or peers, a relaxed style of the provider that avoids interrogation and aligns the tone of feedback with the older adult’s level of concern appeared to be particularly important to engage older adults.^20^,^46^,^124^–^127^ Finally, digital interventions may attract an important subset of older adults who subsequently reduce harm.^111^

**Table:** 

**Facilitators to Successful Prevention Interventions Among Older Adults**
***Macro Factors***
Screening, brief intervention, and referral to treatment (SBIRT) as a reimbursable intervention^66^
Greater societal normalization of discussing alcohol^20^
Community building with alcohol-free activities and events for older adults^20^
Resilience activities at the individual, small group, and wider community level^20^
***Setting***
Places in which older adults may be waiting and available for intervention (e.g., primary care waiting rooms, pharmacy line, hospital atriums, grocery stores)^20^,^54^,^59^,^70^,^127^
Part of a Medicare annual wellness visit^67^
Flexibility in terms of time, location, and frequency of contact^127^
Nonstigmatizing settings, including in home^22^,^23^,^110^,^123^,^126^
***Primary Prevention Intervention Factors: Psychoeducation***
Group education (also provides community as a protective factor)^20^,^53^,^56^,^57^
Gamification^56^
Variety of educational mediums, posters, brochures, and public service announcements^57^,^127^
Sustained education over time (repetition, reminders, booster sessions)^20^
Inclusion of citations of facts to increase trust in the information^63^,^79^,^128^,^129^,^133^,^136^,^137^
***Digital Prevention Intervention Factors***
Normative feedback, especially for older men^79^,^103^
Personalized feedback, especially for older women^28^,^57^,^70^,^81^,^82^,^110^
Gamification^56^,^76^,^83^
A variety of messaging approaches (i.e., not only health-based messages)^68^
Eliciting active engagement, not just passive information^83^,^111^
Inclusion of citations of facts to increase trust in the information^63^,^79^,^128^,^129^,^133^,^136^,^137^
***Other Intervention Factors***
Assessment and feedback using the AUDIT, AUDIT-C, ARPS, or the CARET^22^,^23^,^55^,^65^,^66^,^70^,^82^,^84^,^96^,^97^,^99^,^104^,^108^,^109^,^112^
Physician provides feedback and is part of agreement to reduce drinking^69^,^96^,^98^–^100^,^102^,^125^,^138^
Both physician and patient receive feedback^69^,^70^,^96^,^98^–^100^,^102^,^138^
Printed materials, detailed booklet^54^,^70^
Personalized and normative feedback^55^,^58^,^63^,^70^
Drink tracking, daily diary^45^,^96^,^97^,^99^,^100^,^102^
Agreement to reduce drinking, commitment, accountability^96^,^97^,^99^,^100^,^102^
Age-specific and awareness of relevance to specific participant^53^,^57^,^60^,^127^,^128^,^133^,^136^
Human contact (peers, providers, small groups)^57^,^135^
Encouraging older adults to both provide and receive help^57^,^127^
Motivational interviewing and/or cognitive behavioral therapy^22^,^23^,^81^,^82^,^94^,^96^,^97^
Multiple points of contact over time^96^,^97^,^99^
***Provider Factors***
Easy relaxed style^20^
Avoids interrogation^20^
Aligns feedback tone with older adults’ level of concern for alcohol use^20^
Long established relationships with older adults (particularly for African Americans)^65^,^150^
Nonmedical providers (younger and African American older adults)^65^,^150^

Studies also identified several factors that motivate older adults to sustain or change drinking behavior (see the box “Reasons to Change or Sustain Drinking Among Older Adults”). Like adults across the life span, older adults reported taking pleasure in the ritual of drinking, that drinking adds to their quality of life and socialization, and that it provides relaxation.^128^,^134^ Pleasurable factors potentially unique to older adults were that it is one last pleasure in their lives, connects them to their youth, and makes them feel enjoyably rebellious.^127^,^128^,^134^ Older adults also reported using alcohol to escape or cope with distress related to loss, illness, or lack of purpose, and in some cases as a form of “medicine” to treat pain, reduce stress, and aid sleep.^128^,^133^–^135^,^139^ Older adults reported a willingness to change when it leads to tangible benefits, such as seeing family or travel;^128^,^139^ to avoid negative consequences and stigma;^20^,^134^,^135^,^139^ or when there was a change in their life circumstance that may require change, such as lack of money or presence of a homecare worker who is monitoring drinking.^134^,^140^

**Table:** 

**Reasons to Change or Sustain Drinking Among Older Adults**
***Reasons for Continued Drinking***
Aspects that Older Adults Specifically Report as Rewarding
“The ritual”^44^,^64^
Adds to quality of life, relaxation^128^,^134^,^139^
Important part of later life, last pleasure^44^,^64^
Part of identity as fun, connected to youth^44^,^64^
The rebellious feeling that comes with drinking^108^
Part of Socialization^128^,^134^
Meaningful part of time with friends^128^,^134^,^139^,^152^
Meaningful part of time with spouse or partner^139^
Cultural norms with family and friends, expectation of drinking^134^
Occurs in settings where there are adverse social consequences for not drinking (e.g., in retirement community)^128^,^134^
With meals and on special occasions^128^,^134^
Often in settings with an abundance of alcohol where socializing^128^,^134^
Motivation for Drinking
Marks the structure of the day^134^
Coping
Coping with loss, bereavement^128^,^134^
Coping with illness or cancer (hopelessness)^128^
Coping with loss of purpose^127^
Among men, used to hide degeneration due to aging^128^
“Form of medicine”^128^,^133^–^135^,^139^
Treats pain^133^
Sleep aid^134^
Positive effects on health and well-being^128^,^134^,^135^
Reduces stress^133^,^139^
No Benefits to Reducing Drinking
Optimistic bias: no problems yet; won’t affect me^128^,^134^,^152^
Perceive little risk, view self as controlled, no need to reduce^128^,^134^
Perceive little benefit from acting on risk information^133^,^135^
Habit, perhaps too difficult to break^128^
***Reasons to Control or Stop Drinking***
To Optimize Life
To be able to see and spend time with grandchildren, family commitments^128^,^139^
To be able to travel^128^
Part of a positive view of aging^152^
To Avoid Negative Consequences
Hangovers, blackouts^134^,^139^
Medication interactions^20^
Avoid drunk driving^128^,^134^,^135^
Avoid ill health^128^,^135^,^140^
Fear of falling, looking foolish^128^
Stigma of Heavy Drinking^128^,^134^
Particularly for women^110^,^128^,^134^,^135^
Religious/cultural beliefs^20^,^128^,^134^
Change in Life Circumstance
Not going out as much^140^
Don’t have money^140^
Trying to lose/control weight^128^,^135^
Not able to drink as much due to age^128^
Doctor gave me advice to reduce^128^
Homecare workers encourage not drinking^128^
If became ill, would reduce^139^

## Discussion

This narrative review described primary, secondary, and tertiary alcohol prevention interventions with older adults, the barriers and facilitators to those interventions, and the impact of age, period, and generation. Like prior reviews,^24^,^26^,^28^ design issues and vague intervention descriptions inhibit definitive conclusions. Because this narrative review compared findings across heterogenous study designs, conclusions are inherently limited. In aggregate, however, it identified consistent aspects of successful prevention interventions among older adults and helped to identify gaps in the literature not previously reported.

### Importance of Assessment

The high prevalence of null findings of prevention interventions compared to assessment suggests there was a therapeutic effect of screening alone, particularly when the AUDIT, AUDIT-C, ARPS, or CARET were used.^22^,^23^,^55^,^65^,^66^,^70^,^82^,^84^,^96^,^97^,^99^,^104^,^108^,^109^,^112^ The AUDIT-C focuses on drinking quantity and frequency and, like drink tracking, may raise awareness of alcohol use. The AUDIT, ARPS, and CARET identify alcohol problems, and the older adult-specific ARPS and CARET also bring to light complexities of comorbidity, offering unique opportunities for reflection. Assessment is clearly an instrumental first pass at prevention, and experiential exercises that accompany such assessment, such as asking older adults to pour their usual drink to determine its equivalent in standard drinks,^20^,^127^ are likely impactful across generations.

### Outcomes Across Generations

Alcohol-related targets, such as planning for change, treatment engagement, and treatment completion, were successfully achieved across modalities and levels of intervention intensity. However, the specific quantity and frequency of alcohol use was more immovable, with a few studies demonstrating that a notable proportion of participants continued to drink at at-risk levels. Older generations demonstrated larger reductions in drinking across prevention interventions and decade of age, yet as BB individuals aged and were included in studies, effects appeared muted. For example, only three out of 18 RCTs including BB participants identified significant findings with this group (see Table 2). This does not suggest that BB older adults do not demonstrate therapeutic benefits from prevention intervention. Rather, few studies were able to demonstrate intervention superiority to a control or an active comparison group, and effects among BB participants appear smaller in magnitude than among prior generations. That said, conclusions made about the influence of age, period, and generation should be interpreted with caution, as they are difficult to parse out. It could be that in this era of limitless options for consuming information, educational messages and interventions are simply not able to make the impact they did with earlier generations.

### Aspects of Successful Prevention Interventions

The review identified shared aspects of successful prevention interventions. Successful educational programs were engaging,^56^ occurred individually and in groups,^53^,^59^ and were provided in multimedia formats,^61^,^68^ particularly in environments where multiple sources clamored for attention.^59^,^61^ Messaging content beyond health consequences, such as the gains of reducing alcohol use (e.g., increased energy, clear thinking, and younger appearance), may provide new in-roads to engage older adults.^68^,^111^ Drink tracking, accountability to another person to reduce drinking, and multiple points of contact and/or ongoing support were present in many of the interventions that demonstrated a significant therapeutic effect over the comparison group. Among TSG older adults, drink tracking 7 days before a primary care appointment, committing to change with a physician or nurse, and having follow-up discussions with physicians or health educators were important aspects for two of the largest effective secondary interventions.^96^–^100^,^102^ Interventions that were beneficial among BB older adults included remote support and education without interacting directly with another person,^108^,^111^ perhaps due to their ability to provide increased privacy, anonymity, and an opportunity to change on their own. Approaching discussions of alcohol use as a part of a healthy lifestyle may initially attract older adults’ attention, but direct conversations about alcohol use, including its importance in an older adult’s life, within and outside health care settings, normalizes and destigmatizes alcohol use and increases the chance for optimal information for next steps.

### Older Adult Preferences and Attitudes Toward Interventions

Surprisingly, alcohol use severity was not a factor determining in which type of prevention interventions older adults engaged. Studies of older adults receiving brief treatments found that many participants preferred minimal care^82^ or reduced alcohol use and harm despite not receiving the full intervention.^22^,^23^ In other studies, however, older adults preferred intensive interventions to minimal care.^125^ Cognitive behavioral therapy, an evidence-supported approach to treating older adults with AUD,^30^,^141^ was offered in four studies; however, cognitive behavioral therapy modules were infrequently implemented, with providers or older adults deeming it unnecessary after one session. Cognitive behavioral therapy protocols adapted for older adults also may not address this population’s heterogeneity, as they were primarily tested among GG/TSG male veterans.^21^,^142^,^143^

Matching older adults to the appropriate level of prevention intervention intensity remains a challenge but could be essential for increasing access to treatment, greater engagement, and reduction of alcohol use. Assessing preferences for interventions along with discussions about alcohol could inform providers on how to best align prevention interventions with older adults’ perceptions of their own drinking and their needs.

### Older Adult-Specific Interventions

Prevention intervention effectiveness was moderately related to older adult specificity. Only 67% of older adult specific interventions reported statistically significant therapeutic effects over the comparison or control groups, whereas 47% of non-older adult-specific prevention interventions demonstrated such effects. Older adult specificity may be less important as severity of alcohol use increases and older adults receive evidence-supported treatments, such as cognitive behavioral therapy.^144^,^145^ Notably, older adult specificity is not equivalent to relevance (i.e., does an older adult perceive a need for this information or experience the information as applicable to them?) or tailoring (i.e., adjusting intervention components to the needs of an older adult). Older adult specificity accounts for biology (e.g., physiological changes that occur with aging) and life stage (e.g., retirement) to identify commonalities across older adults. Relevance and tailoring extend beyond this and address the unique role alcohol plays in an older adult’s life. Set and setting of interventions matter across the life span.

### Digital Interventions

Contrary to stereotypes that older adults will not or cannot engage with digital interventions, 64 online and mobile digital interventions were used across generations and decades with promising results.^58^,^63^,^84^ In some cases, older adults engaged twice as often to achieve the same therapeutic effect;^89^ however, a meta-analysis^29^ examining individual-level data demonstrated that older adults age 55 and older had almost twice the odds of responding to online alcohol treatment compared to younger adults. Older adults who seek online help may already view their alcohol use as a concern. In a mediational analysis of online personalized feedback and normative feedback, participants who were already worried about their drinking before feedback had a significantly greater likelihood of planning for change.^63^,^79^,^137^ Assessing a person for concerns about their drinking before intervention may further help providers and programs align interventions with an older adult’s level of concern, thereby increasing engagement.

### Potential Adaptations to Prevention Interventions

#### Primary care as a setting for intervention

The future of primary care prevention intervention is unclear, as primary care providers already shoulder impossible burdens, with one study estimating that it would require 26.7 hours daily to implement all recommended prevention services for a panel of 2,500 primary care patients annually.^146^ Where GG/TSG older adults consistently responded to primary care advice, younger generations did not. Possible explanations are generational attitude shifts away from doctors as the ultimate authority,^147^ as well as shortened primary care visits and high turnover that prohibit long-term relationships with providers. Another factor may be the inclusion of more racial/ethnic minorities in later studies who may distrust research in medical settings. Thus, the infamous Tuskegee syphilis study and other medical abuses^148^,^149^ understandably impact Black older adults’ trust in medical research across generations and may explain why nonmedical interventionists with long-term community-based relationships have greater impact with African Americans.^65^,^150^

#### Expanding FRAMES for older adults

In the 1990s, active components of alcohol brief interventions for all adults were compiled into the FRAMES approach that emphasizes feedback, responsibility, advice, menu of options, empathy, and self-efficacy.^151^ Based on the present review, FRAMES could be updated for older adults to reflect what is known about the potential moderating effects of sex, generation, and alcohol use severity. Normative feedback may be particularly important for older men, who may be more influenced by peers, whereas personalized feedback may be more important for women and younger cohorts of older adults, who may be more responsive to health messages. In addition to representing “advice,” the A of FRAMES for older adults could stand for agreement for change and accountability to another person, whereas the S could stand not only for self-efficacy but also for social or professional support, especially as drinking severity increases.

#### The Health Promotion Workbook for Older Adults

For the past 30 years, most older adult-specific prevention interventions have used Barry et al.’s *Health Promotion Workbook for Older Adults*.^21^ This mainstay of SBIRT adaptations to older adults contributed to reducing alcohol among GG/TSG older adults;^85^,^88^,^99^ however, once BB individuals entered studies of older adults, findings became more variable.^96^ Although workbook elements such as drink tracking and an agreement with another person to reduce drinking remain a part of effective interventions across generations, other aspects of the workbook may now be outdated, especially given advances in aging medicine, generational knowledge, alcohol research, and increasing older adult heterogeneity. Updating the workbook or digitizing it to incorporate artificial intelligence to increase relevance and tailoring to each older adult may be a promising avenue for future intervention and research. This could also widen the reach of these interventions in a cost-effective way to rural areas, where providers and treatment opportunities are scant.

### The Drink Wise, Age Well Initiative

“Drink Wise, Age Well” (DWAW) was a public health initiative implemented in the United Kingdom from 2015 to 2020 to address alcohol use among individuals age 50 and older.^127^ DWAW included more than 40 interventions at the macro, mezzo, and micro levels, harnessing multipronged best practices for prevention intervention. Strategically constructed, adapted, and implemented with local community partners, DWAW recorded extensive positive impact in its final report, which was published but not peer reviewed.^127^ DWAW included education; peer-to-peer activities; trainings; brief interventions^20^ and brief treatments,^127^ one of which was reviewed above; a dedicated help line to connect individuals to formal treatment; and more.

Within this initiative, Live Wise, Age Well (LWAW)^127^ was a six-module, older adult-specific cognitive behavioral therapy group focused on resilience and finding purpose. When outcomes of LWAW participants were compared to those receiving formal specialty care, LWAW demonstrated equivalent alcohol outcomes yet retained older adults longer. Among LWAW participants (*N* = 2,466), 24% were no longer showing at-risk drinking patterns, 74% improved well-being, 45% reduced anxiety, and 44% reduced depression. Of note, DWAW worked with community partners from project inception and prioritized community building, social connections, and a sense of purpose among older adults by providing tailored interventions for local communities. Implementing prevention at the macro, mezzo, and micro levels and DWAW’s broad focus on individual and community resilience, community building, and alcohol-free activities, which may inherently lead to reduced alcohol use, are compelling features that merit future rigorous empirical study.

### Future Policy, Research, and Conclusion

Despite the limitations of existing research, the future of prevention interventions aimed at older adults is bright. Investment of time and resources into this burgeoning group could increase engagement of this population in brief interventions by disseminating existing prevention interventions that include assessment and the identified components of successful interventions described above, as well as honor the perspectives and reasons for change among older adults. Expanding ongoing education about alcohol use in later life for individuals, providers, and communities could contribute to reduced individual and societal harm. Future policy could address structural components that inhibit provider engagement in SBIRT^151^ and extend and deepen providers’ professional development to increase competency with older adults. Future research could test common features of successful interventions identified in this review—including intensity, messaging content, and attention to providers and relationships—as well as new additions, such as an updated workbook or a rigorous test of LWAW. Refinement of older adult prevention interventions that isolates their active components and updates education for new generations may enhance implementation and effectiveness. Finally, findings highlight the utility of offering older adults a variety of prevention intervention modalities, such as in-person, online, and mail-based approaches, to increase access and engagement across generations, honoring older adults’ heterogeneity and their self-determination.

**KEY TAKEAWAYS**
- Older adults born from 1924 to 1945 (The Silent Generation) had strong responses to primary, secondary, and tertiary alcohol prevention interventions, particularly those that involved a physician (i.e., a physician delivered the intervention or followed up regarding alcohol use), with multiple contacts over time, and across decades of life. However, Baby Boomers did not respond as well or as consistently to the interventions.
- Intervention components that showed effectiveness across intervention types, decades of life, and generations included drink tracking, discussion with another person about alcohol, an agreement to change, and aligning the tone of the provider with the older adult’s level of concern about their own drinking.
- Digital interventions showed promise across decades of age and generations, including web- and text messaging-based interventions. This finding is important given that primary care may no longer be an ideal setting for interventions for older adults due to the high burdens placed on providers and other barriers.
- A large proportion of studies found that screening and/or assessment alone were as effective as the active interventions they were testing, particularly when screening/assessment was conducted using older adult-specific tools, such as the Alcohol-Related Problems Survey and its descendant, the Comorbidity-Alcohol Risk Evaluation Tool. Other non-older adult-specific instruments that produced similar effects were the Alcohol Use Disorder Identification Test and the Alcohol, Smoking, Substance Involvement Test.

## Figures and Tables

**Table 1:** Characteristics of Included Primary Prevention Studies to Reduce Alcohol Use, Secondary Prevention Studies to Prevent Development of Alcohol Use Disorder, and Tertiary Prevention Studies to Prevent Worsening of Alcohol Use Disorder or Associated Complications

Characteristics	Primary Prevention Studies *(n* = 21) *n (%)*	Secondary Prevention Studies (*n* = 25) *n (%)*	Tertiary Prevention Studies (*n* = 5) *n (%)*	Total Studies (*N* = 51) *n (%)*
***Country***				
United States	16 (76.2)	17 (68.0)	4 (80.0)	37 (72.5)
United Kingdom	3 (14.3)	3 (12.0)	0 (0)	6 (11.8)
Australia	0 (0)	2 (8.0)	0 (0)	2 (3.9)
Denmark	0 (0)	2 (8.0)	0 (0)	2 (3.9)
China	1 (4.8)	0 (0)	0 (0)	1 (2.0)
Croatia	1 (4.8)	0 (0)	0 (0)	1 (2.0)
Spain	0 (0)	1 (4.0)	0 (0)	1 (2.0)
Sweden	0 (0)	0 (0)	1 (20.0)	1 (2.0)
***Demographics***				
Generations included				
GG/TSG	5 (23.8)	9 (36.0)	1 (20.0)	15 (29.4)
GG/TSG/BB	0 (0)	0 (0)	1 (20.0)	1 (2.0)
TSG only	2 (9.5)	6 (24.0)	0 (0)	8 (15.7)
TSG/BB	11 (52.4)	7 (28.0)	2 (40.0)	20 (39.2)
TSG/BB/Gen X	3 (14.3)	1 (4.0)	1 (20.0)	5 (9.8)
BB only	0 (0)	2 (8.0)	0 (0)	2 (3.9)
BB/Gen X	1 (4.8)	1 (4.0)	0 (0)	2 (3.9)
24% or more non-White	5 (23.8)	8 (32.0)	2 (40.0)	15 (29.4)
50% or more female	14 (66.7)	7 (28.0)	3 (60.0)	24 (47.1)
***Study Design***				
Pre-to-posttest	12 (57.1)	4 (16.0)	0 (0)	16 (31.4)
Randomized comparison trial or quasi comparison design	1 (4.8)	1 (4.0)	2 (40.0)	4 (7.8)
Randomized controlled trial	8 (38.1)	20 (80.0)	3 (60.0)	31 (60.8)
***Setting (not mutually exclusive)***				
Primary care	9 (42.9)	14 (56.0)	1 (20.0)	24 (47.1)
Specialty clinic for disease/injury	1 (4.8)	2 (8.0)	0 (0)	3 (5.9)
Hospital/emergency department/in-patient setting	3 (14.3)	1 (4.0)	1 (20.0)	5 (9.8)
Community, senior centers	6 (28.6)	4 (16.0)	1 (20.0)	11 (21.6)
Outpatient research setting	0 (0)	1 (4.0)	2 (40.0)	3 (5.9)
Website/online	3 (14.3)	5 (20.0)	0 (0)	8 (15.7)
Pharmacy	1 (4.8)	0 (0)	0 (0)	1 (2.0)
***Modality (not mutually exclusive)***				
Mail	1 (4.8)	2 (8.0)	0 (0)	3 (5.9)
Booklet, pamphlet, report	3 (14.3)	2 (8.0)	0 (0)	5 (9.8)
In person (group or one-on-one)	11 (52.4)	18 (72.0)	5 (100)	34 (66.7)
Online, video conference	5 (23.8)	3 (12.0)	0 (0)	8 (15.7)
Telephone	1 (4.8)	6 (24.0)	0 (0)	7 (13.7)
Computer in office	1 (4.8)	1 (4.0)	0 (0)	2 (3.9)
Mobile or smartphone	0 (0)	3 (12.0)	0 (0)	3 (5.9)
***Design/Adaptation***				
Older-adult specific	15 (71.4)	16 (64.0)	2 (40.0)	33 (64.7)
***Approach (not mutually exclusive)***				
Psychoeducation	10 (47.6)	3 (12.0)	0 (0)	13 (25.5)
Motivational interviewing	2 (9.5)	11 (44.0)	5 (100)	18 (35.3)
Brief alcohol intervention/SBIRT	5 (23.8)	8 (32.0)	2 (40.0)	15 (29.4)
Brief advice	1 (4.8)	7 (28.0)	0 (0)	8 (15.7)
Brief negotiated interview	2 (9.5)	0 (0)	1 (20.0)	3 (5.7)
Cognitive behavioral therapy	0 (0)	3 (12.0)	2 (40.0)	5 (9.8)
Goal setting	1 (4.8)	2 (8.0)	2 (40.0)	5 (9.8)
Normative or personalized feedback	0 (0)	5 (20.0)	3 (60.0)	12 (23.5)
Text messaging	4 (19.0)	2 (8.0)	0 (0)	2 (3.9)
Daily self-monitoring	0 (0)	3 (12.0)	0 (0)	3 (5.9)
Review of workbook	4 (19.0)	8 (32.0)	0 (0)	12 (23.5)
Physician involvement	3 (14.3)	3 (12.0)	0 (0)	6 (11.8)
Other	0 (0)	4 (16.0)	3 (60.0)	7 (13.7)
***Intervention Characteristics***				
Only one contact	20 (95.2)	6 (24.0)	0 (0)	26 (51.0)
Multiple contacts	1 (4.8)	19 (76.0)	5 (100)	25 (49.0)
Contact time less than 1 hour	5 (23.8)	7 (28.0)	0 (0)	12 (23.5)
Contact time 1 hour or more	3 (13.6)	11 (44.4)	5 (100)	19 (37.3)
Time not reported	12 (61.9)	8 (28.0)	0 (0)	20 (39.2)
Not possible to track (mail, text)	1 (4.8)	1 (4.0)	0 (0)	2 (3.9)
***Types of Outcomes (not mutually exclusive)***				
Knowledge only	6 (28.6)	0 (0)	0 (0)	6 (11.8)
Intention/plan to change only	4 (19.0)	0 (0)	0 (0)	4 (7.8)
Completed screen	2 (9.5)	0 (0)	0 (0)	2 (3.9)
Treatment engagement or completion	0 (0)	1 (4.0)	1 (20.0)	2 (3.9)
Alcohol use (quantity/frequency)	10 (47.6)	25 (100)	3 (60.0)	38 (74.5)
Reduced harm (i.e., at-risk drinking)	12 (57.1)	25 (100)	3 (60.0)	40 (78.4)
Discharge to home	0 (0)	0 (0)	1 (20.0)	1 (2.0)
***Intervention Achieved Target Goal***				
Knowledge only	6/6 (100)*	0/0 (0)*	0/0 (0)*	6/6 (100)*
Intention/plan to change only	4/4 (100)*	0/0 (0)*	0/0 (0)*	4/4 (100)*
Completed screen	2/2 (100)*	0/0 (0)*	0/0 (0)*	2/2 (100)*
Treatment engagement or completion	0/0 (0)*	1/1 (100)*	1/1 (100)*	2/2 (100)*
Reduced quantity/frequency of alcohol	4/10 (40.0)*	13/25 (52.0)*	3/3 (100)*	20/38 (65.8)*
Statistically significant proportion reduced at-risk drinking	7/12 (58.3)*	23/25 (92.0)*	2/2 (100)*	32/39 (87.1)*
Discharge to home from skilled nursing facility	0/0 (0)*	0/0 (0)*	1/1 (100)*	1/1 (100)*

* Percentages are by row, rather than column.

*Note:* More than one option could apply to a study, so values do not necessarily add up to 100%. BB, Baby Boomers; Gen X, Generation X; GG, Greatest Generation; RCT, randomized controlled trial; SBIRT, Screening, Brief Intervention, and Referral to Treatment; TSG, The Silent Generation.

**Table 2:** Randomized Controlled Trials of Interventions with a Significant Effect on Target Outcome Over the Comparison or Control Group by Age, Period, and Generations Included

Ages by Decade	Study*	Years Data Collected	Predominant Generation Represented†	Type of Prevention Intervention
50–60	Copeland et al. (2003)^69^	1994–1999	TSG	Primary
	Oslin et al. (2003)^88^	2000–2001	TSG	Secondary
	Kuerbis et al. (2023)^45^	2008–2009, 2012–2016	BB	Tertiary
	Kuerbis et al. (2015)^108^	2011–2012	BB	Secondary
	Kuerbis et al. (2022)^111^	2014–2015	BB	Secondary
61–70	Fleming et al. (1999)^85^	1993–1995	TSG	Primary
	Copeland et al. (2003)^69^	1994–1999	TSG	Primary
	Oslin et al. (2003)^88^	2000–2001	TSG	Primary
	Bartels et al. (2004)^90^	2000–2001	TSG	Secondary
	Zanjani et al. (2008)^93^	2000–2001	TSG	Secondary
	Wooten et al. (2017)^95^	2000–2001	TSG	Secondary
	Lee et al. (2009)^94^	2001–2005	TSG	Secondary
	Moore et al. (2011)^96^	2004–2007	TSG	Secondary
	D’Agostino et al. (2006)^123^	NR‡	TSG	Tertiary
	Ettner et al. (2014)^99^	2005–2007	TSG	Secondary
	Kuerbis et al. (2023)^45^	2008–2009, 2012–2016	TSG/BB	Tertiary
	Kuerbis et al. (2015)^108^	2011–2012	TSG/BB	Secondary
71–80	Fleming et al. (1999)^85^	1993–1995	GG	Primary
	Bartels et al. (2004)^90^	2000–2001	TSG	Secondary
	Zanjani et al. (2008)^93^	2000–2001	TSG	Secondary
	Lee et al. (2009)^94^	2001–2005	GG/TSG	Secondary
	Wooten et al. (2017)^95^	2000–2001	TSG	Secondary
	Fink et al. (2005)^70^	2000–2003	GG/TSG	Primary
	Moore et al. (2011)^96^	2004–2007	TSG	Secondary
	Ettner et al. (2014)^99^	2005–2007	TSG	Secondary
	D’Agostino et al. (2006)^123^	NR‡	TSG	Tertiary

* Some studies fell into more than one age decade.

† Defined as the generation that covered the widest span of participants according to age (mean +/− 1 SD) and years of data collection.

‡ Used date of publication and year before.

*Note:* BB, Baby Boomers; GG, Greatest Generation; TSG, The Silent Generation.

**Appendix 1:** Characteristics and Outcomes of the Primary Prevention Interventions in Older Adult Populations Included in This Review (*N* = 21)

Study (Country) Project Name	*N* Year Data Collected Generation	Sample Population Age (Range or Mean + SD) Race Sex	Setting Modality Older Adult-Specific (OAS)	Intervention	Comparator	Outcome
***Within Group Designs (Pre-to-Post)***						
Eliason et al. (2001)^53^ (United States)	*N = 26* NR GG/TSG	Healthy volunteers Mean age: 75.1 +8.5 100% White 100% female	Community, senior center In person OAS	1-hour group education on metabolism of alcohol and drugs, medication interactions, and healthy lifestyle after age 60	NA	Significant within-group increase in knowledge of drug and alcohol use at immediate follow-up
Fink et al. (2001)^54^ (United States)	*N* = 101 NR GG/TSG	Primary care patients Age: 60+ 25% non-White 57% female	Primary care Booklet / pamphlet OAS	Education materials about alcohol and aging (36-page booklet or pamphlet) in primary care waiting room	NA	Both booklet and pamphlet significantly improved within-group knowledge; booklet was preferred
Nguyen et al. (2001)^55^ (United States)	*N* = 106 NR GG/TSG	Primary care patients age 60+ who drink any alcohol Mean age: 72.2 + NR 8% Hispanic; 3.8% non-White 51.5% female	Primary care Computer OAS	Computerized ARPS completed in waiting room of primary care, included feedback to participant and health education	NA	At immediate follow-up, 31% intended to talk to their physician about the report. People with harmful drinking levels were more likely to intend to discuss with physician.
Benza et al. (2010)^56^ (United States) Prevention BINGO	*N = 348* 2007 TSG/BB	Healthy volunteers Age: 50+ 78% female	Community, senior center In person OAS	Psychoeducation via Prevention BINGO game with small prizes; education about co-use of medications and alcohol	NA	Significant increases in knowledge of risks of alcohol use at 30-day follow-up
Zanjani et al. (2012)^57^ (United States)	*N* = 55 2010–2011 TSG/BB	Healthy volunteers Mean age: 65.4 + NR; median age 65 85% female	Community; cooperative extension site in rural Kentucky In person OAS	1-hour group education and training (what to do when seeing someone in need and how to intervene); awareness campaign (bookmarks, pamphlets, note pads with strategies for promoting and managing mental health)	NA	Significant improvement in awareness at 3 months, sustained at 6 months; knowledge at 3 months, partially sustained at 6 months. At 6 months, lost knowledge around risks for older adults who do not drink much and other misperceptions.
Han et al. (2018)^58^ (United States)	*N* = 78,663 2013 TSG/BB	Website visitors who reported unhealthy drinking Age 21–49: *n* = 60,976 Age 50–65: *n* = 15,316 Age 66–80: *n* = 2,371 41% female	Visitors to alcoholscreening.org website Online Not OAS	Personalized feedback about risks and sex-based normative feedback; assistance in developing a plan to change	NA	Of the three age groups, the two older groups and females had higher odds of receiving a plan for change at immediate follow-up compared to those under age 50
Zanjani et al. (2018)^59^ Zanjani et al. (2020)^62^ (United States)	*N = 134* 2015–2016 TSG/BB	Patients at rural pharmacies Mean age: 71.6 + 7.5 63.4% female For individuals who completed 3-month follow-up: Mean age: 72.1 + 3.7 60% female	Pharmacy In person OAS	Psychoeducation via informational poster, brochure, and 1-minute public service announcement; could ask research assistant about materials and take as long as they like to review them	NA	Knowledge significantly increased at immediate follow-up, but there was no change in intention to discuss alcohol medication interactions with pharmacist or change drinking. People who drank any alcohol were more willing to discuss and change drinking. At 3-month follow-up, people with high-risk drinking patterns reduced drinks by half (15 to 7 per week). Medication side effects significantly decreased among all people who drank.
Kuerbis et al. (2017)^63^ (United States)	*N* = 138 2016 BB/Gen X	Age: Range 50–70, with only two participants age 65+ 46% female	Online labor market Online OAS	Normative feedback (percentile rank of drinking among peers, based on age and sex); personalized feedback (level of risk based on the CARET and safe guidelines according to their level of risk)	NA	Immediately post-intervention, level of worry predicted plan for change. Those receiving personalized feedback were significantly more surprised by feedback than those receiving normative feedback. Belief in the accuracy of feedback was significantly improved when given citations for feedback. About 44% planned for change, regardless of feedback type.
Seddon et al. (2024)^20^ (United Kingdom) Drink Wise, Age Well	*N* = 3,999 2015–2020 TSG/BB/Gen X	Healthy volunteers Age: 50+ 1% non-White British 58% female	Supermarkets, lobbies, shopping centers, stations In person OAS	SBIRT with FRAMES	NA	Among people with hazardous drinking (i.e., drinking beyond recommended low-risk drinking) (*n* = 2,726), 41% reported intention to change drinking immediately after the intervention.
Scott et al. (2020)^65^ (United States)	*N* = 302 NR TSG/BB	Primary care patients Mean age: 72.7 + 8 95% African American, 99% non-White 73% female	Primary care, community In person OAS	Brief negotiated interview, motivational interviewing, assessed readiness, prescription for change, referral information; no primary care	NA	Alcohol use was reduced. Fewer individuals screened positive for alcohol use disorder using AUDIT-C and DAST at each follow-up, 52% positive at 3 months and 35% at 6 months.
Brown et al. (2014)^66^ (United States)	*N = 675* 2020–2021 TSG/BB	Universal screening Age: 12% were 65+, CTYA 37% non-White, 7% Hispanic 59% female	Primary care, emergency department, hospital, tribal clinic In person Not OAS	ASSIST, SBIRT, or brief intervention, including motivational interviewing or referral to specialty treatment; no primary care involved	NA	Across all ages, 20% reduction in at-risk drinking, as measured by ASSIST. People age 65 and older showed the lowest response. Only 11% change in proportion of people with at-risk drinking at 6 months.
Tarn et al. (2023)^67^ (United States)	*N = 1,513* 2021 TSG/BB	Medicare patients Mean age: 71.5 + 11.3 7% non-White 57% female	Primary care Video conference OAS	Annual wellness visit (AWV); addition of templates for use of EHR tools, supporting tasks, and improving efficiency; video conference with provider	NA	Significant increase in completion of AWV. Among people completing the AWV, alcohol screening significantly increased and was three times the rate of people who did not complete the AWV at 12 months.
***Randomized Comparison Trials***						
Sallis et al. (2019)^68^ (United Kingdom)	*N = 104,227* 2014 TSG/BB/Gen X	All website visitors Age: 33% age 45+, CTYA 48% female	Drink-aware website Online Not OAS	Psychoeducational messages to engage in AUDIT-C screen and pursue further resources; appearance vs. health messages	NA	Appearance messages led to more AUDIT-C completion among older adults age 65 and older. Among adults ages 35–64, health framing led to accessing further resources.
***RCTS***						
Copeland et al. (2003)^69^ (United States) Healthy Profiles Study	*N* = 205 1994–1999 TSG	Male veterans Mean age: 66.1 + 6.4 24% Black	Primary care In person OAS	Brief alcohol intervention booklet with overview by primary care physician	General health advice booklet with overview by primary care physician	Alcohol outcomes were not reported. The intervention increased specialty substance use outpatient treatment at 9 months; there were no differences in other care utilization and no difference at 18 months.
Fink et al. (2005)^70^ (United States) A Toast to Health	*N* = 665 2000–2003 GG/TSG	Primary care patients Mean age: 76.6 + 6.2 53% female	Primary care Printed report OAS	Personalized feedback and alcohol education given to patient; report given to patient and primary care provider	Usual care	Computerized ARPS was used to assess drinking outcomes. The group that received only the patient report had 59% greater odds, and the group that received the combined report had 23% greater odds, to drink at low-risk levels compared to usual care at 12 months.
Harari et al. (2008)^71^ (United Kingdom)	*N = 2,006* 2001–2002 GG/TSG	Community-dwelling people age 65+ Mean age: 74.4 + 6.1 54% female	Primary care Printed report OAS	Mailed results on the multidomain Health Risk Appraisal for older persons; 25–30 pages feedback sent to patient (and physician), cover letter suggesting they speak with their doctors	Usual care	No difference between groups on alcohol outcomes, only on receiving vaccines and physical activity at 6-month and 12-month follow-up.
Vrdoljak et al. (2014)^72^ (Croatia)	*N = 738* 2008–2010 TSG	Primary care patients age 65+ Mean age: 72.3 + 5.2 61% female	Primary care In person OAS	Primary care physicians trained to treat a variety of health behaviors including alcohol misuse; physicians implemented treatment at their discretion; health booklet to follow, with patient leaflets	Leaflet on cardio-vascular health	No group differences except for greater shift toward Mediterranean diet. No changes in alcohol use at 18-month follow-up.
Wolinsky et al. (2017)^73^ Cram et al. (2016)^74^ (United States)	*N* = 7,749 2012–2014 TSG/BB	Individuals age 50+ receiving a bone density scan Mean age: 66.6 + 8.3 25% non-White 84% female	Bone density scan clinic Mailed letter Not OAS	DXA scan results mailed with a health educational letter 4 weeks after scan; letter advised on health behaviors related to smoking and excessive alcohol use	Usual care	For two outcome questions: Do you drink alcohol? and What is the typical amount you drink each day? there was no effect of the letter at 3 or 12 months.
Shenvi et al. (2022)^75^ (United States)	*N* = 98 2012–2015 TSG/BB	Patients age 65+ presenting to emergency department Mean age: 73 + NR 17.3% non-White 32% female	Emergency department In person OAS	Brief intervention that (1) related alcohol to emergency department visit; (2) compared person’s drinking to low-risk guidelines; (3) identified readiness to change; (4) asked for person to develop goal	Usual care	No difference existed between groups at 6 months. Both groups significantly reduced at-risk drinking at 6-month follow-up compared to baseline drinking.
Fink et al. (2016)^76^ (United States) A Toast to Health Online	*N = 96* 2014 TSG/BB	People age 55+ Mean age: 70 + NR 68% female	Community Online OAS	Psychoeducation via a website with information about health, dangerous behaviors, drinking, and quantity	No intervention	Drinking measured by ARPS and quantity/frequency of drinking found no difference between the groups. Both groups decreased drinking by 2 drinks. Only the *perception* of reduced drinking was significant among the intervention group 4 weeks after the website visit.
Wan et al. (2016)^77^ (China)	*N* = 80 2014 TSG/BB/Gen X	Stroke patients, 12.5% younger than age 44 Mean age: 59.7 + 12.5 29% female	Hospital In person, telephone Not OAS	Pre-discharge education followed by three 15–20-minute goal-setting calls at 1 week, 1 month, and 3 months after discharge	Pre-discharge education only	There was no difference between groups on alcohol use. Medication adherence exhibited the only significant difference at 3 months and 6 months.

*Note:* ARPS, Alcohol Related Problems Survey; ASSIST, Alcohol, Smoking, Substance Involvement Test; AUDIT, Alcohol Use Disorders Identification Test; AUDIT-C, Alcohol Use Disorders Identification Test-Consumption; BB, Baby Boomers; CARET, Comorbidity-Alcohol Risk Evaluation Tool; CTYA, compared to younger adults; DAST, Drug Abuse Screening Test; FRAMES, Feedback, Responsibility, Advice, Menu of options, Empathy, and Self-efficacy; Gen X, Generation X; GG, Greatest Generation; NA, not applicable; NR, not reported; OAS, older adult-specific; RCT, randomized controlled trial; SBIRT, Screening, Brief Intervention, and Referral to Treatment; TSG, The Silent Generation.

**Appendix 2:** Characteristics and Outcomes of the Secondary Prevention Interventions: Preventing Alcohol Use Disorder (AUD) Included in This Review (*N* = 26)

Study (Country) Project Name	*N* Year Data Collected Generation	Sample Population Age (Mean + SD, Median) Race Sex	Setting, Modality, Older Adult-Specific (OAS)	Intervention	Comparator	Outcome
***Within Group Designs (Pre-to-Post)***						
Schonfeld et al. (2010)^23^ (United States) Florida BRITE Project, Pilot	*N = 3,497* 2004–2007 GG/TSG	People age 60+ with positive screen for substance use Mean age: 74.9 + 9.2 24% non-White 69% female	Community, agencies serving seniors In home, in person OAS	SBIRT, guided by OAS health promotion workbook; one to five 1-hour sessions with an option for CBT within those sessions	NA	Specific to alcohol: Of subsample with follow-up data, 82% were no longer positive (2+) at discharge on SMAST-G.
Schonfeld et al. (2015)^22^ (United States) Florida BRITE Project	*N = 85,001* older adults screened using ASSIST; 8,165 were positive; 516 with follow-up data. 2006–2011 TSG/BB	People age 55+ who screened positive for alcohol misuse as measured by ASSIST Mean age: 66.5 + 8.9 21% Hispanic, 31% non-White 39% female	Agencies serving seniors, in homes, and health care settings as required by SAMHSA grant In person OAS	SBIRT, guided by OAS health promotion workbook, with an option to use cognitive behavioral therapy for up to 16 sessions (few utilized)	NA	Thirty-three percent follow-up rate for those who were eligible (out of 516). At 6 months, proportion with any alcohol use (measured via ASSIST) was reduced from 75% to 30% and intoxication was reduced from 47% to 23%. About 10% no longer reported binge drinking.
Bright and Williams (2017)^81^ Bright and Williams (2018)^82^ (Australia) Older Wiser Lifestyles (OWL)	*N = 140* Before 2013 TSG	Individuals age 60+ who drank alcohol in past month Mean age: 72.8 + 7.6 46% female	Community, primary care Online, in person OAS	Screening online or in primary care using the Australian version of the ARPS (A-ARPS); session with nurse/psychologist tailored to stage of change as indicated by the Contemplation Ladder: low risk = minimal education; at-risk and precontemplation = 1 hour session on risks, psychoeducation; contemplation = brief intervention, FRAMES, one to five 1-hour sessions with motivational interviewing and goal setting; functional analysis, problem solving was optional	NA	The A-ARPS was used to measure at-risk drinking. Of participants with hazardous or harmful drinking patterns, 54% received only minimal intervention (because they thought it was enough). Minimal intervention and brief intervention significantly reduced AUDIT-C scores. Mean consumption was still above the recommended limit, except among those who received minimal intervention (i.e., who had started with lower drinking levels). The proportion of people with harmful drinking was reduced from 68% to 36% regardless of intervention at the 3-month and 6-month follow-up.
Bolt et al. (2024)^83^ (Australia)	Pre-to-post *N* = 289 2020–2021 BB	People with at-risk drinking patterns with desire to reduce drinking, age 55+ Median age 60, Interquartile range 57–63; 61% female	Community Smartphone app Not OAS	Approach bias modification training was provided within an app on a personal smartphone. Training occurred 2 times per week for 4 weeks.	NA	TLFB measured drinking. Low-risk drinking increased significantly from 25% to 41% for those receiving single session and from 6% to 28% for those receiving weekly guidelines. The mean reduction in weekly drinking was 5.43 standard drinks. Significant reductions in craving and dependence symptoms were noted at 4-week follow-up.
***Randomized Comparison Trials***						
Sedotto et al. (2024)^84^ (United States)	*N* = 105 2020 BB/Gen X	People age 50+ recruited on Facebook who screened positive on the AUDIT Mean age: 58.5 + 6.5, CTYA 24% non-White 55% female	Online smart-phone app; Facebook messaging with chatbot Not OAS	*Step Away* app with eight modules, including drink tracking; assessment and feedback; goal setting; social supports; coping with craving; strategies for moderation/abstinence; reminders of motivation; new activities and more help	Chatbot delivered via Facebook without relying on user initiation; daily check-in	Outcomes were determined using AUDIT, SIP, TLFB, RTCQ. No significant differences in alcohol use, SIP, and RTCQ across age groups; all were significantly reduced. Older adults used *Step Away* twice as much as young adults, reported it more usable, used it for more hours and total days as well as at the 3-month follow-up.
***RCTS***						
Fleming et al. (1999)^85^ Mundt et al. (2005)^86^ (United States) Project Guiding Older Adult Lifestyles (GOAL)	*N = 158* 1993–1995 GG/TSG	People age 65+ with at-risk drinking 33% female	Primary care In person OAS	Two 10- to 15-minute counseling sessions with primary care, included brief advice, education, and contracting using a booklet; sessions were 1 month apart	General health booklet	Outcomes were assessed using TLFB, drinks/week, binge episodes, excessive drinking, and cost. Significant reductions occurred for all alcohol measures for the intervention group compared to control. Differences were maintained at 24 months. There were no economic gains from the reduced use.
Gordon et al. (2003)^87^ (United States) Early Lifestyles Modification Study	*N* = 171 1995–1997 GG/TSG	People age 65+ (*n* = 45) with at-risk drinking and CTYA 31% non-White 3% female	Primary care In person Not OAS	Motivational enhancement therapy, goal setting (one session, 45–60 minutes, two 15-minute boosters at 2 and 6 weeks); or brief advice (one 15-minute session)	Usual care	All conditions significantly decreased drinking over time. No differences existed between conditions or age groups. Despite reductions in drinking, average consumption showed substantial at-risk drinking at 12 months as measured by TLFB.
Oslin et al. (2003)^88^ (United States)	*N = 97* 2000–2001 TSG	Veterans with depression and/or at-risk drinking Mean age: 61.6 + 10.5 50% non-White 4% female	Primary care Telephone OAS	Telephone disease management (TDM), calls conducted at 1, 3, 6, 9,12, and 24 weeks after baseline; at-risk drinking was addressed with OAS workbook	Usual care	Outcome was measured by TLFB. At 4 months, TDM showed significant improvement over usual care with treatment response (44% vs. 20%), treatment access (41% vs. 10%), and drinks per week (reduced by 9 units vs. 2 units) and binge episodes reduced (26 vs. 1). Treatment access was not associated with treatment response.
Oslin et al. (2004)^89^ (United States) UPBEAT	*N = 2,637* 1995–1998 GG/TSG	Hospitalized veterans age 60+ with depression, anxiety, or at-risk drinking Mean age: 69.7 + 6.6 29% non-White 3% female	Hospital, Veterans Administration Medical Center In person, telephone OAS	Comprehensive, interdisciplinary assessment by team; assignment of case manager to coordinate, reduce barriers to care; tailored to the individual; duration for as long as needed	Usual care	There were no differences between groups. There was improvement on overall health, mental health functioning, physical functioning, and AUDIT scores, which decreased about seven points by 24 months.
Bartels et al. (2004)^90^ (United States) PRISM-E	*N* = 2,022 2000–2001 GG/TSG	People with at-risk drinking, age 65+ Mean age: 73.5 + 6.2 48% non-White 26% female	Primary care In person OAS	Integrated care (IC) = behavioral mental health care targeting alcohol use provided in primary care, within 2 to4 weeks; brief alcohol intervention based on FRAMES and workbook	Enhanced referral to specialty care	Greater treatment engagement was observed in IC at 6 months compared to enhanced referral to specialty care.
Oslin et al. (2006)^91^ (United States) PRISM-E	*N* = 560 2000–2001 GG/TSG	People with at-risk drinking age 65+ Mean age: 72 + 5.3 30% non-White 8% female	Primary care In person OAS	IC = behavioral health provided in primary care; three 20–30-minute brief alcohol counseling sessions with workbook	Enhanced referral to specialty care	For engagement, 43% of IC participants received at least one brief alcohol intervention session; only 9% had all three sessions. Both groups significantly reduced drinks per week from 17 to 11 units. Binge episodes in the last 3 months were reduced from 21 to 11 at the 6-month follow-up.
Zanjani et al. (2008)^93^ (United States) PRISM-E	*N* = 258 2000–2001 GG/TSG	Male veterans age 65+ with at-risk drinking Mean age: 71.6 + 4.6 34% non-White	Primary care In person OAS	IC = behavioral health provided in primary care targeting alcohol use	Enhanced referral to specialty care	In IC: Problem drinkers identified by SMAST-G significantly reduced weekly alcohol consumption and binge drinking compared to non-problem drinkers at 12 months. Those randomized to IC had higher treatment engagement. Twenty-nine percent of all participants sustained at-risk drinking at 12 months.
Lee et al. (2009)^94^ (United States) PRISM-E	*N = 34* 2001–2005 GG/TSG	People age 65+ with at-risk drinking Mean age: 74.9 + 8.0 59% non-White; 61% female	Primary care In person OAS	IC = behavioral health provided in primary care, harm reduction; three motivational interviewing sessions	Enhanced referral to specialty care	IC participants were more likely to access care and receive services sooner. At 6 months, significant within (IC only) and between-group differences existed in drinks per week and binge episodes per 3 months; no differences existed on SMAST-G.
Wooten et al. (2017)^95^ (United States) PRISM-E	*N* = 438 NR GG/TSG	Male veterans age 65+ with at-risk drinking Mean age: 71.9 + 5.0 28% non-White	Primary care In person OAS	IC = behavioral health provided in primary care; the most effective site additionally had comprehensive, specialty geriatric care and an interdisciplinary team	Enhanced referral to specialty care	At 6 months, at-risk drinking was reduced by half in both groups. Only one clinic had a significant difference—people in the IC group were 75% less likely to exhibit at-risk drinking.
Moore et al. (2011)^96^ Lin et al., (2010)^97^ (United States) Healthy Living as You Age (HLAYA)	*N* = 631; 310 (received health educator calls) 2004–2007 TSG	People age 55+ with at-risk drinking Mean age: 68.4 + 6.9 9% Hispanic, 13% non-White 29% female	Primary care In person, Telephone OAS	Personalized feedback given to patient and physician; daily diary self-monitoring before primary care visit; physician brief advice with booklet, follow-up health educator calls at 2, 4, and 8 weeks, with motivational interviewing; first call was 40 minutes, subsequent calls were 20 minutes	Booklet on general health behaviors alone	People with at-risk drinking who received intervention reduced risk measured by CARET at 3 months, which was sustained at 12 months but did not differ significantly from booklet only. At 12 months, there was a significant difference in drinks per week. The odds of being at low risk at 3 months were 5.3 in people who had received all three calls vs. no calls. There was no effect at 12 months.
Ettner et al. (2014)^99^ Duru et al. (2015)^100^ Barnes et al. (2010)^101^ Barnes et al. (2016)^102^ (United States) Project Share	*N = 1,186* 2005–2007 TSG	People with at-risk drinking Mean age: 71 + 7.3 6% Hispanic, 3% non-White 34% female	Primary care In person, mail, telephone OAS	Mailed psychoeducation, personalized feedback, booklet, tip sheets; physician to talk about alcohol at every visit over the year; three health educator calls (~27 minutes total) at 2 weeks, 3 months, and 6 months	Usual care	Intervention was significantly better on multiple outcomes at 6 months and 12 months measured by CARET, including at-risk drinking, drinks per week, discussion with primary care provider, and health care utilization. Odds of at-risk drinking at 6 months were .58 for those with drinking diary or agreement, and .52 for those with both. Odds of at-risk drinking at 12 months were .61 for those who had had discussions with the primary care provider and .45 for those with both diary and agreement.
Gottlieb Hansen et al. (2012)^103^ (Denmark)	*N = 1,380* 2008–2010 TSG/BB	People with heavy drinking (females above 14 standard drinks/week, males above 21 standard drinks/week) Median age: 58, IQR NR for total sample 45% female	Community Online Not OAS	Personalized feedback/normative feedback vs. brief advice vs. control; Web-based personalized feedback (with sex-based normative feedback); personalized brief advice (risks of heavy drinking, advice to cut down only), or control	None, only screen with their name	There were no group differences. All groups reduced drinking an average of 6 standard drinks per week by 12 months. In a subgroup analysis, men appeared to respond significantly better to the personalized feedback intervention, compared to the other groups.
Gottlieb Hansen et al. (2012)^44^ (Denmark)	*N = 772* 2008–2009 TSG/BB	People with at-risk drinking Median age: 59, IQR: 50–65 49% female	Research participants in a health examination In person, telephone Not OAS	Brief motivational intervention lasting 11 minutes, handout on alcohol and available treatment, 5-minute booster call 4 weeks later	Handout only	No group differences in the quantity or frequency of alcohol use at 12 months. Reported poor quality of motivational interviewing measured by Motivational Interviewing Treatment Integrity (MITI) scale.
Watson et al. (2013)^104^ Coulton et al. (2017)^105^ (United Kingdom) AESOPS	*N = 529* 2006–2010 TSG/BB	People age 55+ with at-risk drinking Mean age: 63 + 5.8 20% female	Primary care In person OAS	Stepped care with 20 minutes behavioral counseling, followed by three 40-minute motivational enhancement therapy sessions, then referral to local specialist	5-minute brief advice with nurse	There were no significant differences; both groups reduced drinking as measured by AUDIT-C at 12 months.
Cucciare et al. (2013)^106^ Cucciare et al. (2013)^107^ (United States)	*N* = 167 2010–2011 TSG/BB	Veterans with at-risk drinking Mean age: 59 + 10 8% Hispanic, 12% Black, 11% other non-White 12% female	Veterans Administration primary care In person Online OAS	Usual care plus primary care referring patients to web-delivered normative feedback about alcohol, tailored to age and sex	Usual care	At 12 months, both conditions reduced alcohol use; percentage of heavy drinking days was reduced by half by follow-up, drinking intensity by about one standard drink per day, three fewer days per month, and 2–3 points lower on SIP. Normative feedback reduced depression (PHQ-9) and increased approach coping (post-traumatic stress disorder checklist).
Kuerbis et al. (2015)^108^ (United States)	*N* = 86 2011–2012 TSG/BB	People age 50+ drinking beyond FDA low-risk drinking guidelines Mean age: 64.7 + 8.4 9% Hispanic, 2% non-White 34% female	Mail, recruited from primary care network Mailed booklet OAS	Mailed brief feedback intervention, booklet on alcohol and aging, and “Rethinking your drinking” pamphlet	CARET assessment only	At 3 months, the intervention group had fewer people with at-risk drinking (66% vs. 88%); fewer people with binge drinking (45% vs. 68%); and fewer participants using alcohol with medical or psychiatric conditions (3% vs. 17%) or with symptoms of those conditions (29% vs. 49%).
Wilson et al. (2014)^109^ (United Kingdom)	*N* = 172 2011–2012 TSG/BB	People with at-risk drinking and with hypertension or depression Mean age: 64 + 9.2 25% female	Primary care In person OAS	Personalized and normative structured feedback on alcohol with tips for reducing drinking; duration 5 minutes	None	At 6 months, both groups lowered AUDIT scores; the hypertension group reduced AUDIT scores about 1 point more than control. For the depression group, there was no difference between conditions.
Torán et al. (2023)^110^ (Spain)	*N = 225* 2012–2015 TSG/BB	People age 65+ with at-risk drinking Mean age: 71 + 5 55% female	Primary care or in home In person OAS	Brief alcohol intervention consistent with WHO recommendations at four time points: initial appointment, 1 month, 6 months, and 12 months.	Minimum advice at initial visit	Reduction to low-risk drinking was considered a success; decreased drink-ing that exceeded low-risk levels was considered partial success. Effects were only significant among women; they were 3.6 times more likely than men to achieve full or partial success, largely explained by abstinence at 12 months.
Kuerbis et al. (2022)^111^ (United States)	*N* = 151 2014–2015 BB	People age 50+ from the general population with at-risk drinking 55.4 + 4.2, CTYA 3% Hispanic, any race, 10% non-White 73% female	Alcohol-related help-seeking websites Text messaging Not OAS	Text message conditions were theoretically framed in four ways: gain, loss, static, and adaptive; daily text messages encouraging reduced drinking for 12 weeks; assessment occurred once weekly.	Assessment only	At 3 months, all text message groups reduced drinking vs. assessment only. Younger adults had slightly larger effects than older adults. Depression, craving, and problems were reduced for gain-framed messages for older adults, loss-framed messages for younger adults. Despite decreases of 5 units per week and one less drinking day per week, a large proportion maintained at-risk drinking.
Snowden et al. (2020)^112^ (United Kingdom)	*N* = 68 2016–2018 TSG/BB/Gen X	Individuals age 40–85, undergoing elective hip or knee arthroplasty, and reporting at-risk drinking Mean age: 66.2 + NR, median age 67 19% female	Hospital, preoperative assessment clinic In person, Telephone Not OAS	Session 1 included AUDIT; 5 minutes of brief advice; and 15 to 20 minutes of behavioral counseling with goal setting, problem-solving, increasing social support handouts; an optional session 2 with a 20-minute booster was conducted 1 week before surgery to review progress and self-efficacy	Usual care	At 6 months, both groups significantly reduced their proportion of people with at-risk drinking measured by AUDIT. No condition differences were found.

*Note:* ARPS, Alcohol Related Problems Survey; ASSIST, Alcohol, Smoking, Substance Involvement Test; AUDIT, Alcohol Use Disorders Identification Test; AUDIT-C, Alcohol Use Disorders Identification Test–Consumption; BB, Baby Boomers; CARET, Comorbidity-Alcohol Risk Evaluation Tool; CTYA, compared to younger adults; DAST, Drug Abuse Screening Test; FDA, U.S. Food and Drug Administration; FRAMES, Feedback, Responsibility, Advice, Menu of options, Empathy, and Self-efficacy; Gen X, Generation X; GG, Greatest Generation; IQR, interquartile range; NA, not applicable; NR, not reported; OAS, older adult-specific; PHQ-9, 9 item Patient Health Questionnaire; RTCQ, Readiness to Change Questionnaire; RCT, randomized controlled trial; SBIRT, Screening, Brief Intervention, and Referral to Treatment; SIP, Short Inventory of Problems; SMAST-G, Short Michigan Alcohol Screening Test–Geriatric version; TLFB, Timeline Followback; TSG, The Silent Generation.

**Appendix 3:** Characteristics and Outcomes of the Tertiary Prevention Interventions for Alcohol Use Disorder and Its Consequences Included in This Review (*N* = 6)

Study (Country) Project Name	*N* Year(s) Data Collected Generation	Sample Population Age (Range or Mean + SD) Race Sex	Setting Modality Older Adult-Specific (OAS)	Intervention	Comparator	Outcome
***Quasi Comparison Designs***						
Kuerbis et al. (2013)^46^ (United States)	*N = 38* 2000–2009 GG/TSG/BB	24 people age 54–59 and 14 people age 60+ 0% non-White; 9% female	Community, outpatient research clinic In person Not OAS	Brief treatments: four sessions motivational interviewing or nondirective listening, 12 sessions motivational interviewing plus CBT	No treatment	Drinking was measured with TLFB. At 8 to 12 weeks, older adults with no treatment decreased drinking by 23%. The most consistent decreases in drinking were in older adults who received motivational interviewing plus CBT (37% to 59% decrease).
Cimarolli et al. (2018)^121^ Cimarolli et al. (2021)^126^ (United States) Geriatric Substance Abuse Recovery Program	*N* = 271 NR TSG/BB	People age 55+ with alcohol use disorder / substance use disorder Mean age: 68.1 + 8.3 19% Hispanic 49% non-White 35% female	Skilled nursing facility In person, telephone OAS	After assessment, care plan for during and after skilled nursing facility stay; services included counseling, family therapy, and AA; referral to community supports, calls, and home visit for support	Refused participation in program	Odds of discharge to home were 3.2 times higher for those in the recovery program. At 30 days post-discharge, up to 69% reported not relapsing.
***RCTS***						
D’Agostino et al. (2006)^123^ (United States) Geriatric Addictions Program (GAP)	*N* = 99 NR TSG	People age 51+ with substance use and mental and physical health problems Mean age: 73.7 + 8.8 59% female	Within community-based agency, in-home In person OAS	Multidimensional approach: geriatric care management assessment, motivational counseling, combination of aging services and substance use treatment linkages	Traditional referral directly to specialty services	At about 4 months, inpatient completion was 80% in the intervention group and 57% in the control group; outpatient completion was 40% in the intervention group and 10% in the control group.
Wallhed Finn et al. (2018)^125^ Wallhed Finn et al. (2020)^124^ (Sweden)	N = 288 2013–2015 TSG/BB/ Gen X	Individuals who met ICD-10 criteria for alcohol dependence Mean age: 55 + 11.3 45% female	Primary care, community specialty care clinic In person Not OAS	“15-method”; 30-minute brief advice with physician, choice of medication and/or brief treatment (four 15-minute sessions of motivational interviewing and cognitive behavioral therapy)	Physician feedback, medication (30 minutes/visit), and/or brief treatment, 45-minute sessions with psychosocial treatment (four sessions of motivational enhancement therapy, guided self-change, behavioral self-control; six to eight sessions of relapse prevention, or 12-week 12-step facilitation)	Both groups increased the proportion of low-risk drinking by 13% in primary care and 19% in specialty care, but this was nonsignificant. Clients reported higher satisfaction in specialty care. Moderation by age group was not significant. By 12 months, most participants in both groups had reduced alcohol consumption by one WHO risk level.
Kuerbis et al. (2023)^45^ (United States) Project Motion	*N* = 228 2008–2009 2012–2016 TSG/BB	Adults age 51+ with alcohol use disorder and moderation goal Mean age: 57.9 + 5.7; CTYA 14% Hispanic, 11% Black 55% female	Outpatient research clinic In person Not OAS	Four 1-hour sessions of either motivational interviewing or nondirective listening (focused on emotions)	Self-change condition	TLFB was used to measure drinking. At 8 weeks, older adults in motivational interviewing reduced drinking 8% more than those in nondirective listening; there was no difference with self-change. Changes were statistically significant but not clinically meaningful. Younger adults did best in nondirective listening.

*Note:* AA, Alcoholics Anonymous; ASSIST, Alcohol, Smoking, Substance Involvement Test; AUDIT, Alcohol Use Disorders Identification Test; AUDIT-C, Alcohol Use Disorders Identification Test–Consumption; BB, Baby Boomers; CARET, Comorbidity-Alcohol Risk Evaluation Tool; CBT, cognitive behavioral therapy; CTYA, compared to younger adults; Gen X, Generation X; GG, Greatest Generation; NR, not reported; OAS, older adult-specific; RCT, randomized controlled trial; SBIRT, Screening, Brief Intervention, and Referral to Treatment; SIP, Short Inventory of Problems; TLFB, Timeline Followback; TSG, The Silent Generation; WHO, World Health Organization.
